# Supplementary material for: A paintbrush for delivery of nanoparticles and molecules to live cells with precise spatiotemporal control
Source: Nat Methods. 2024 Feb 12;21(3):512–20. doi: 10.1038/s41592-024-02177-x (PMC10927540; doi:10.1038/s41592-024-02177-x)
Supplement: Supplementary file 1 — Supplementary Sections 1–11, including Supplementary Figs. 1–24. [file 41592_2024_2177_MOESM1_ESM.pdf]

nature methods

Article

https://doi.org/10.1038/s41592-024-02177-x

A paintbrush for delivery of nanoparticles and molecules to live cells with precise spatiotemporal control

In the format provided by the authors and unedited

Contents

1 Micropipette Fabrication, Geometry & Performance **2**

1.1 Micropipette Fabrication 2

1.2 Micropipette Hydrodynamic Resistance 4

1.3 Experimental Arrangement of the Micropipettes 4

1.4 Numerical Simulation (COMSOL) 6

1.5 Experimental Positioning of the Micropipettes 7

1.6 Positioning to μkiss on a Sample 8

1.7 Micropipette and Envelope Stability and Reproducibility 8

1.7.1 Micropipette Variability 8

1.7.2 Micropipette & Envelope Stability 8

1.7.3 Reproducible Labeling 12

2 μkiss Envelope Velocimetry **14**

3 Characterization of the Flow Envelope **15**

3.1 Parameterizing Envelope Extent 15

3.2 Reynolds Number at the Envelope Boundary 16

3.3 Péclet Number at the Envelope Boundary 16

4 Cell Viability Under μkiss Operation **19**

5 Temporal Response of μkiss Brushing **22**

6 μkiss & FRAP: Analysis & Modeling **23**

7 Sub-cellular Delivery Targeting the Microtubule Network **23**

8 Manders' Coefficient of Tf-AF647:Clathrin Labeling **27**

9 Quantifying TfR Labeling **27**

10 Latrunculin Insult **29**

11 Reagent Delivery from a Single Sub-micron Micropipette **31**

1 Micropipette Fabrication, Geometry & Performance

1.1 Micropipette Fabrication

Fabrication of micropipettes out of a heat-pulled glass capillary is straightforward and has been routinely practiced in laboratories since its inception nearly 50 years ago[1]. In many cases, micropipettes can also be purchased from various vendors. Our method of fabrication is outlined in the following. Borosilicate glass capillaries with an outer diameter $\varnothing_{\mathrm{out}} = 1$ mm and inner diameter $\varnothing_{\mathrm{in}} = 0.78$ mm (GB100TF-10, Science Products GmbH) were cleaned in a series of 10 min sonication steps, first in 2 % Hellmanex, followed by double-distilled water and ethanol. Capillaries were then dried using a heating plate and plasma-cleaned afterwards. Before pulling, capillaries are incubated in 37 % HCl overnight, rinsed with double-distilled water and dried, then plasma-cleaned prior to pulling. A Sutter P-2000 Micropipette Fiber Puller is used to draw the capillaries into the desired micropipettes using the program given in Table 1. The program is completed in three lines.

Heat	Filament	Velocity	Delay	Pull
400	5	200	255	0

Table 1: Pull settings to draw micropipettes with a high-aspect ratio taper and tip-$\varnothing_{\mathrm{in}} = 1\,\mu$m using a P-2000 Sutter Fiber Puller.

The drawn micropipettes are shown in Fig. 1a-c and feature a high-aspect ratio taper, denoted L (Fig. 1a) and $\varnothing_{\mathrm{in}} = 1\,\mu$m at the tip (Fig. 1b,c). The capillaries feature an in-built filament (marked with arrowhead in Fig. 1c) which aids with back-filling of the micropipette through capillary forces.

To accommodate the micropipette on a microscope where the imaging objective occupies the sample space, the tapered length of the micropipette is angled with respect to the main shaft. To introduce this angle, the taper undergoes additional working with a focused CO_2 laser, shown in Fig. 1d. The pulled micropipette is securely mounted within a grip head (Eppendorf) and positioned within the focus of the laser using a micropositioner such that the focus is a length L away from the tip. The laser is operated in pulsed mode, which instantly softens the glass in the focal volume, causing the taper to self-bend towards the laser source, shown in Fig. 1d,e. A stop rod in the path of the bending tip fixes the desired angle θ to which the tip will bend. With no stop, the tip can bend to a full $\theta_{\mathrm{max}} = 90°$.

For micropipettes with similar forms, but larger apertures ($\varnothing_{\mathrm{in}}$), commercially available pipettes from Eppendorf are used (Piezo Drill Tip M.ICSI micropipettes, referred to here as ICSI for short). These micropipettes are characterized by inner diameters of $\varnothing_{\mathrm{in}} = 6\,\mu$m, $L = 6$ mm and $\theta = 25°$, shown in Fig. 1f-h. Owing to the larger

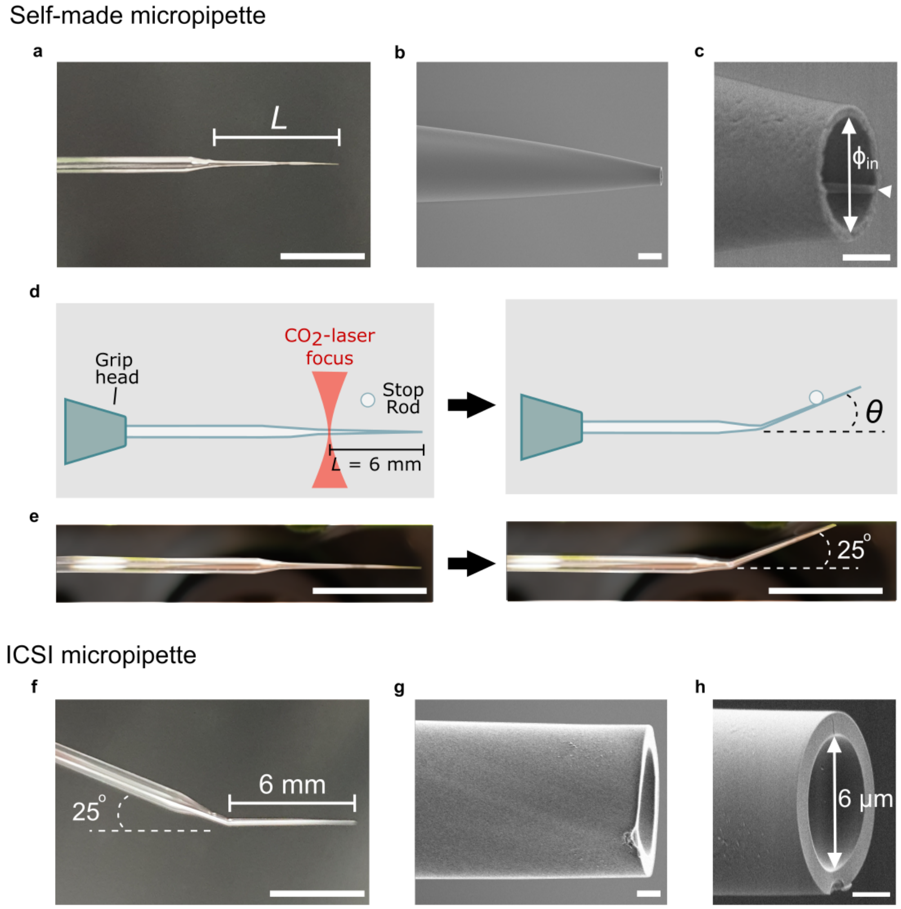

Figure 1: Micropipette fabrication and geometrical form. **a**, Photograph showing a pulled micropipette with a long, thin taper of length $L = 6\,\text{mm}$. Scale bar 5 mm. **b** and **c**, SEM images of the micropipette shown in (**a**), visualizing the very end of the tip taper (**b**) and the micron-sized aperture (of diameter $\varnothing_{\text{in}} = 1\,\mu\text{m}$) with built-in filament (**c**). Scale bars in (**b**) and (**c**) $1\,\mu\text{m}$ and 500 nm, respectively. **d,** Schematic visualization of the process to introduce an angle to the taper. The pulled micropipette is mounted into a grip head and positioned into the focus of a CO_2-laser, with the focus a distance $L = 6\,\text{mm}$ from the tip. Upon heating, the taper bends towards the laser source and a stop rod fixes the angle to θ. **e**, Photographs showing a pulled micropipette before and after the angle-forming process, here with $\theta = 25°$. Scale bar 5 mm. **f**, Photograph showing an Eppendorf ICSI micropipette with $L = 6\,\text{mm}$ and $\theta = 25°$. Scale bar 5 mm. **g, h,** SEM images of the micropipette shown in (**f**), highlighting the linear taper towards the very tip (**g**) and the aperture detail (**h**), wherein $\varnothing_{\text{in}} = 6\,\mu\text{m}$. Scale bars in (**g**) and (**h**) $1\,\mu\text{m}$ and $2\,\mu\text{m}$, respectively.

inner diameter of the micropipettes, a filament is not required and the micropipettes can be filled using a microloader micropipette.

1.2 Micropipette Hydrodynamic Resistance

When fully-developed laminar flow is confined to a channel, one can approximate that rate of fluid flow (Q) is linearly proportional to the pressure drop across the channel (Δp): a scenario which applies to our micropipettes. The constant of proportionality is the hydrodynamic resistance ($\mathrm{R_H}$):

$$\mathrm{R_H} = \frac{\Delta \mathrm{p}}{\mathrm{Q}} \tag{1}$$

The hydrodynamic resistance $\mathrm{R_H}$ is a function of the geometry of the channel, as well as the viscosity of the fluid in the channel and the nature of the slip-condition between the fluid and channel wall, and serves as a useful characterization of the micropipette performance. This is most evident with the reciprocal of hydrodynamic resistance, known as hydrodynamic conductance $\mathrm{C_H}$, which reports on the resultant fluid flow rate within the micropipette as a function of applied air pressure.

We measure the hydrodynamic resistance of our ICSI micropipettes in both injecting and aspirating operation. We do so on the same micropipette to avoid inter-micropipette variation in the characterization. All pressures refer to that which is applied by the pressure regulator (Elveflow OB3+). To determine the fluid flow rate in or out of the micropipette, which is in the $\mathrm{nl\,s^{-1}}$ range, we firstly fill the micropipette with a fluorescein solution to aid visibility ($1\,\mu\mathrm{g}\,\mu\mathrm{l}^{-1}$ fluorescein solution from sodium salt, Merck, 518-47-8). We apply a constant pressure and image the position of the moving solution meniscus over time. With knowledge of the internal geometry of the channel, the shift in meniscus position can be computed as a change in the internal solution volume, which we average over time assuming steady-state operation. Repeating for a range of applied pressures, one can calculate the corresponding induced flow rate - shown in Fig. 2.

1.3 Experimental Arrangement of the Micropipettes

As illustrated in Fig. 3a and b, the μkiss device consists of two similar micropipettes placed adjacent to one another, such that fluid flow out of one tip is subsumed within the inward flow of the second. This is accomplished by positioning the two tips side-by-side so that their apertures lie in the same plane (Fig. 3). The tapers of the two micropipettes lie at an angle of $\theta_{xy} = 35°$ in the mutual plane of their alignment. There is no other relative angle between the two tips in other planes ($\theta_{yz} = \theta_{xz} = 0°$). Small offsets between the contacting outer walls of the tips in the xy plane, denoted $x_{\mathrm{separation}}$ and $y_{\mathrm{separation}}$ in Fig. 3 are introduced to aid flow confinement and adjust brush shape. Typically $x_{\mathrm{separation}} = 1\,\mu$m and $y_{\mathrm{separation}} = 0 - 2\,\mu$m. No offset is introduced in the z-direction, $z_{\mathrm{separation}} = 0\,\mu$m. This arrangement of micropipettes is used throughout this work, depicted in Fig. 3c.

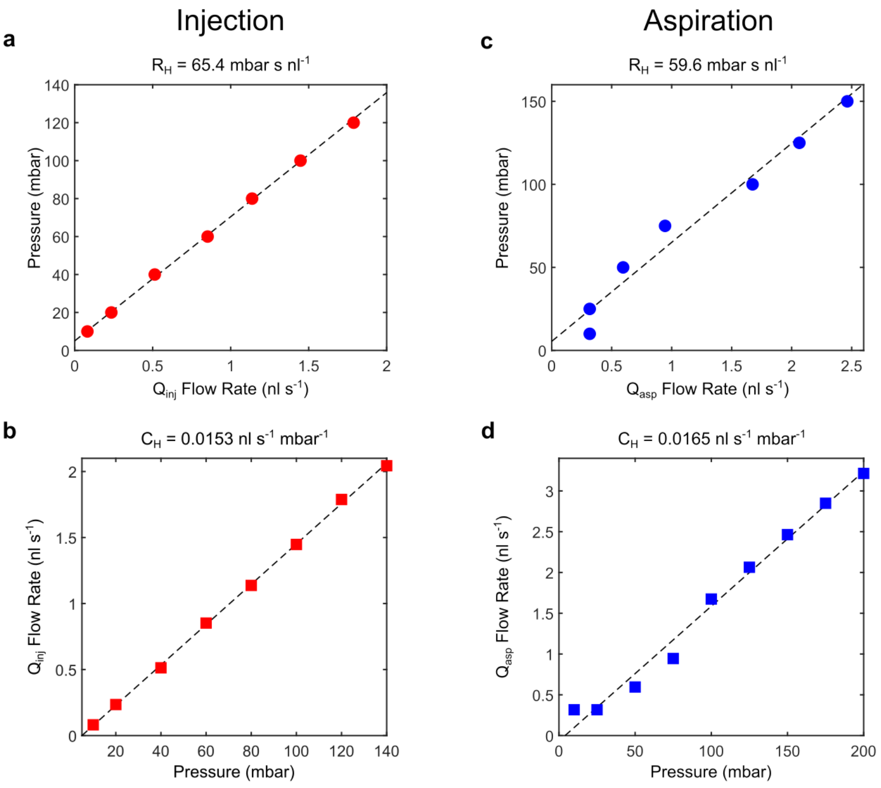

Figure 2: Hydrodynamic resistance and conductance of an ICSI micropipette under both injection and aspiration operation. **a** and **b**, under application of positive pressure (injection operation) the hydrodynamic resistance (**a**) and conductance (**b**). **c** and **d**, Under application of negative pressure (aspiration operation) the hydrodynamic resistance (**c**) and conductance (**d**).

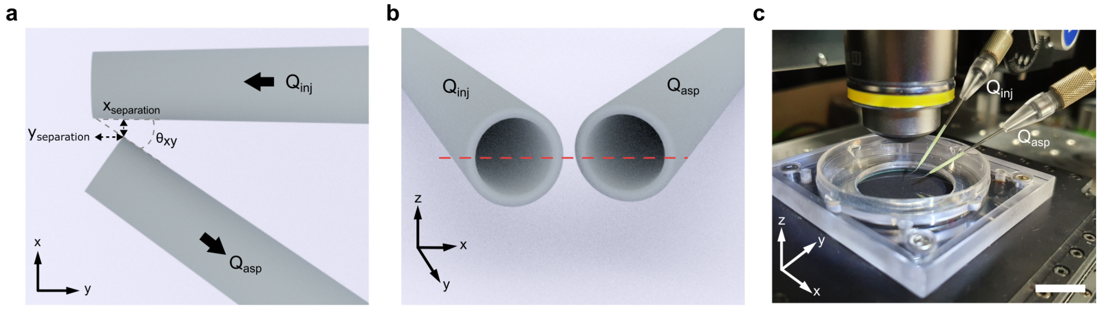

Figure 3: Aligning two micropipettes for μkiss brushing. **a**, Illustration of the spatial arrangement of the two micropipette tips, as viewed top-down (xy plane). **b**, Alternate perspective from (**a**), showing the apertures are aligned, with no out-of-plane offset or rotation. **c**, Photograph showing two micropipettes forming the μkiss device *in-situ* within a cell-bearing dish and with an imaging objective above. Scale bar 1 cm.

Description	Parameter	Value	Unit
Flow rate injected	Q_{inj}	0-2	$\text{nl}\,\text{s}^{-1}$
Flow rate aspirated	Q_{asp}	0-2	$\text{nl}\,\text{s}^{-1}$
inner diameter	$\varnothing_{in}$	6	μm
wall thickness	wall_thick	1	μm
separation in x	x_separation	0	μm
separation in y	y_separation	1	μm
separation in z	y_separation	1	μm
angle in x-y	θ_{xy}	35	degree
angle in y-z	θ_{yz}	0	degree
angle in x-z	θ_{xz}	0	degree

Table 2: Parameters and their values used for modeling the μkiss envelope in COMSOL.

1.4 Numerical Simulation (COMSOL)

COMSOL Multiphysics 6.0 (COMSOL, AG) is used to numerically solve the Navier-Stokes equation for single-phase laminar flow for the dual micropipette geometry. The modeled geometry is shown in Fig. 4 and is modeled with an extra fine Physics-controlled mesh (generated by COMSOL) for both stationary and time-dependant solutions. The single phase flow module (spf module) is used, with physical and geometric parameters given in Table 2. Modeling is performed in full in three dimensions as well as in two dimensions for the xy-plane subtending the centers of both micropipettes, illustrated in red in Fig. 4c for clarity.

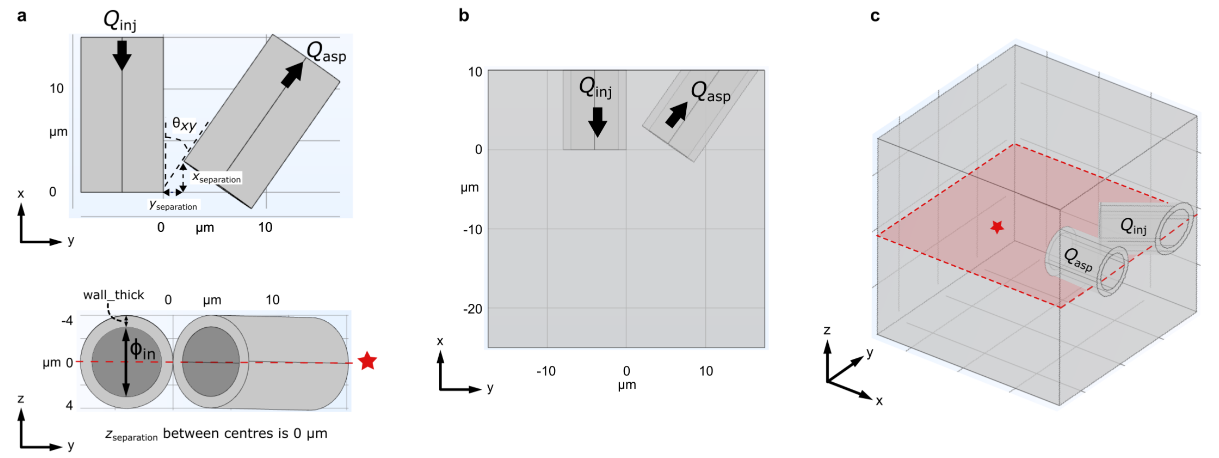

Figure 4: Schematics showing the COMSOL modeling geometry for the μkiss apertures. **a**, xy- and zy-projections of the two tip sections with geometrical parameters marked. Red dashed line marks a plane subtending the two centres of the tips in the xy-plane. **b**, xy-projection of the tips with encompassing 'open-environment' cubic domain shown. **c**, The complete domain shown in (**b**), shown in full three-dimensional projection and with the mid-plane introduced in (**a**) marked for clarity.

The micropipette apertures are modeled within an encompassing cubic domain filled with water, representing

the open fluidic bath environments. A boundary condition at the cube walls assumes the net pressure remains static at environmental pressure and permits flow in and out of the domain. Borosilicate glass is the material assumed for all micropipette glass surfaces and a no-slip condition asserted at glass-water boundaries. Boundary conditions for micropipette walls assert the glass is non-permeable to water. To visualize transport of dissolved species, the transport of dilute species module (tds module) is used, with transport via convection enabled. The velocity field used to compute transport is that solved in the previous step by the spf module for the same geometry. Boundary conditions assume the aperture where Q_{inj} enters is the only source of the dilute species and that the aperture where Q_{asp} is defined also acts as a sink of the dilute species in addition to all the walls of the encompassing cubic domain. No flux of the dilute species occurs through the glass walls, and the initial concentration of dilute species is null.

1.5 Experimental Positioning of the Micropipettes

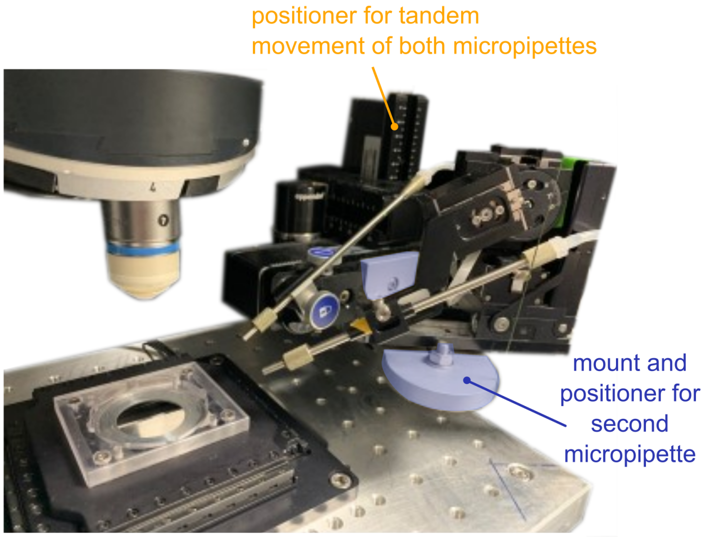

Figure 5: Positioning the micropipette-pair. Photograph of the setup showing the positioner for tandem movement of both micropipettes, as well as the mount on which the positioner of the second micropipette is located (shown in blue highlight).

The micropipette-pair is positioned to sub-micron accuracy by use of two motorized microscale positioners, shown in Fig. 5. The second positioner, to which the aspiration micropipette is mounted, is itself mounted upon a table (shown in blue) to the first positioner. Thus, both micropipettes can be steered in perfect tandem by use of the first positioner alone. For alignment of the second micropipette with respect to the first, the former is used. The positioning of the micropipettes is performed through iSCAT microscopy imaging of the pair *in-situ*. By observation of the interference fringes that are formed when the micropipettes reach within several microns of the glass coverslip surface, one can accurately align the micropipettes along the axial direction with sub-micron accuracy.

1.6 Positioning to μkiss on a Sample

Positioning the μkiss apertures vertically involves placing the micropipette-pair at a height above the target surface such that, upon the establishment of the flow envelope, reagent transfer occurs between envelope and surface. This is readily achieved when imaging with a water-dipping objective by one of two methods. One approach is to first bring the target surface into focus, then raising the objective up a distance slightly larger than the aperture diameter of the μkiss apertures tips. The μkiss apertures, initially situated far away from the target surface, is lowered towards the surface until the full-width of the aperture is in sharp focus. The apertures should now lie close to the target surface without having made contact. The focus can be returned to the plane of the target surface and labeling can begin.

A second approach is to begin with the μkiss apertures running, with envelope established, at a height far above the target surface. With the imaging plane set to the target surface, the μkiss apertures can be lowered progressively until the lower side of the envelope begins to enter the focal depth of the objective (here, the depth of focus is *ca.* $0.75\,\mu$m) and then lowered further until the desired intersection between envelope and surface (that is, brush-stroke area) is achieved.

1.7 Micropipette and Envelope Stability and Reproducibility

1.7.1 Micropipette Variability

The micropipette-to-micropipette variability in aperture size was quantified through SEM imaging of a series of commercially available (ICSI) micropipettes, with 10 exemplar images shown in Fig. 6a. We find the variability in the aperture diameter to be $6.6 \pm 0.5\,\mu$m from the sample of 10 micropipettes, summarized in Fig. 6b. To visualize the extent to which this variation could affect the geometry of the flow envelope, we performed numerical modeling of two scenarios wherein both micropipettes posses the lower bound of the aperture size ($6\,\mu$m) and also the upper bound ($7\,\mu$m), shown in Fig. 6c. For this modeling, we used $Q_{\text{inj}} = 0.15\,\text{nl}\,\text{s}^{-1}$ and $Q_{\text{ratio}} = 0.18$. For clarity, we overlay both envelopes to illustrate the variation in envelope size, which amounts to an approximate change of 900 nm. Considering that the distance between the micropipette-pair and the target surface is adjusted *in-situ* and under surveillance through the microscope, this variability has no consequence.

1.7.2 Micropipette & Envelope Stability

To quantify positional instability of the micropipettes on our apparatus, firstly addressing the short timescale response, we imaged the micropipette ends (see a representative confocal iSCAT [2,3] frame in Fig. 7a) and performed image registration on temporally subsequent pairs of frames for each micropipette in turn (acquisition time for each frame was 50 ms). In doing so, we find a mean positional variation of 101 nm. This operation was performed for when flow was actuated within the micropipette - shown in Fig. 7b, as well as when flow was ceased - shown in Fig. 7c. The strong similarity of these two cases lets us conclude that instabilities caused by compliance of the thin capillaries are negligible at our typical working flow rates (*ca.* $Q_{\text{inj}} = 0.1\text{-}0.3\ \text{nl}\,\text{s}^{-1}$). To address the stability of the micropipette-pair over longer timescales, we repeated the above measurements over a period of 20 min with an acquisition time for each frame now being 0.931 s, shown in Fig. 7d,e for actuated and ceased flow respectively. We find again a mean positional stability on the order of *ca.* 100 nm, again allowing us to conclude that instabilities

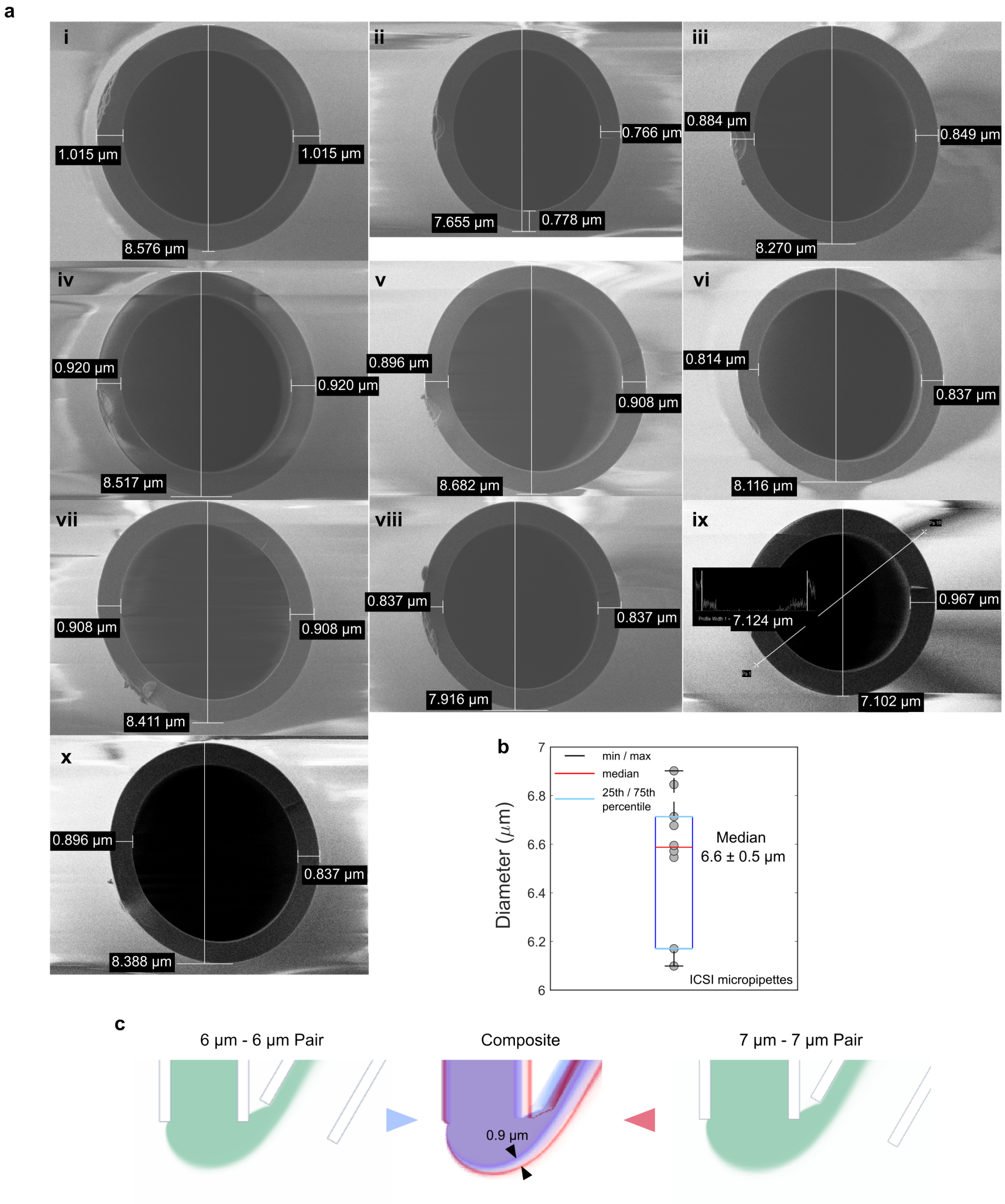

Figure 6: Variability in micropipette dimensions. ICSI aperture variation and effect on flow confinement. **a**,**i-x** 10 SEM images showing exemplary ICSI micropipette openings, indicating the aperture diameters and wall thicknesses. **b**, Box plot of the inner diameter variation among 10 ICSI micropipettes. Median and standard deviation of the distribution are $6.58 \pm 0.51\,\mu$m. The 25th and 75th percentile are $6.17\,\mu$m and $6.71\,\mu$m respectively, the sample minimum and maximum are $5.1\,\mu$m and $6.9\,\mu$m respectively. **c**, Numerical simulations of flow envelopes established by two ICSI micropipette-pairs with $6\,\mu$m and $7\,\mu$m apertures, respectively, as well as a composite image showing the minimal effect of aperture diameter variation on the flow envelope geometry.

are negligible at our typical working flow rates.

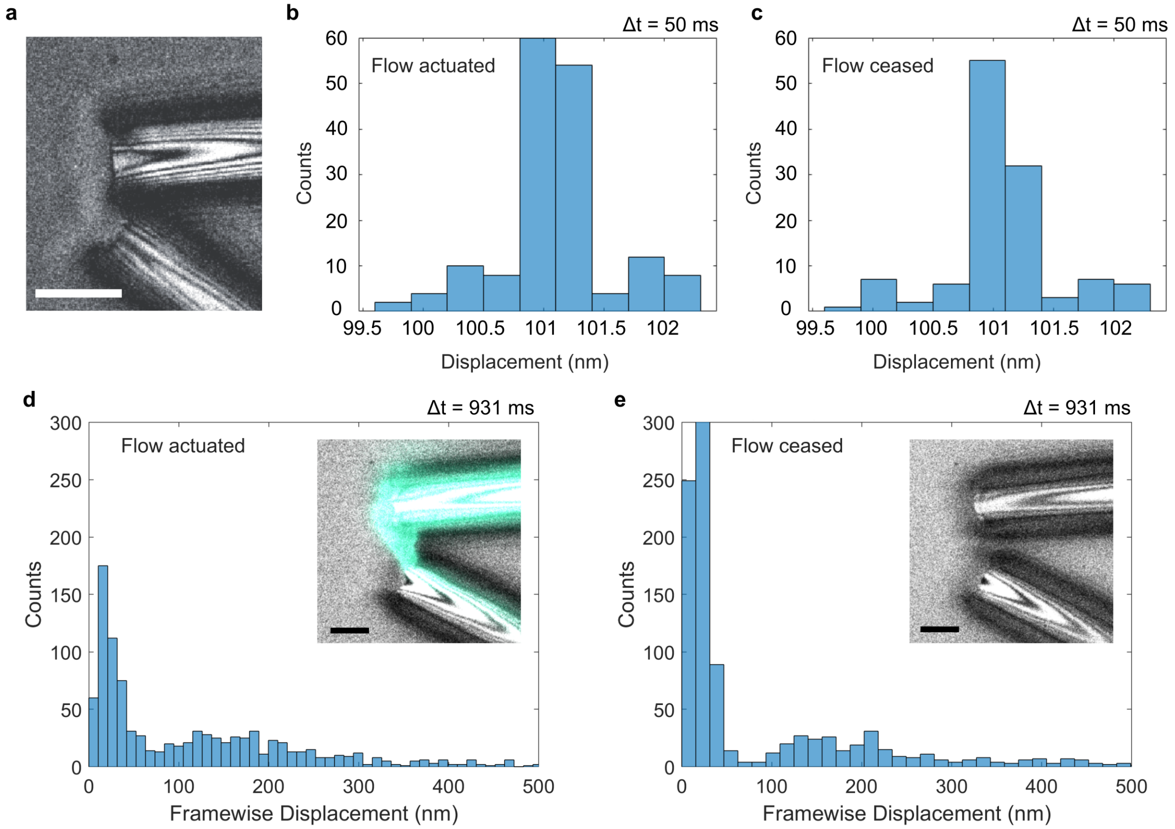

Figure 7: Micropipette positional stability. a, iSCAT image of an ICSI micropipette pair in focus, from which a short timescale time series with frametime of $\Delta t = 50\,\mathrm{ms}$ was recorded to perform image registration to analyze the positional variation. Scale bar is $5\,\mu\mathrm{m}$. The frame-to-frame variation in position of the micropipette, presented in a histogram, for when flow is actuated (**b**) and when flow is ceased (**c**). Here, $Q_{\mathrm{inj}} = 0.16\,\mathrm{nl\,s^{-1}}$ and $Q_{\mathrm{ratio}} = 0.3$. The longer-term positional stability of the micropipette-pair presented as a histogram and recorded with a frametime of $\Delta t = 0.931\,\mathrm{s}$ over 20 min **d**, for when flow is actuated and **e**, when flow is ceased. Inset shows a composite confocal iSCAT (gray scale) and fluorescence (green) image of the micropipette-pair with a fluorescently-labeled reagent under flow. Scale bar is $5\,\mu\mathrm{m}$. Here $Q_{\mathrm{inj}} = 0.07\,\mathrm{nl\,s^{-1}}$ and $Q_{\mathrm{asp}} = 2.96\,\mathrm{nl\,s^{-1}}$.

The variation in the flow envelope boundary position was tracked in time to assess the positional stability of the envelope. To this end, an envelope was established using fluorescein (1 mM solution) as a visual indicator and imaged via confocal fluorescence microscopy for flow rates $Q_{\mathrm{inj}} = 0.15\,\mathrm{nl\,s^{-1}}$ and $Q_{\mathrm{ratio}} = 0.2$ - shown in Fig. 8a. Each captured frame (acquisition time 55 ms) was binarized (cutoff threshold being 5% of the peak signal) and the boundary was mapped - shown in Fig. 8b. The boundary is taken on the region of the envelope exterior to the micropipette channels. We present in Fig. 8c the resultant boundaries extracted from 50 imaging frames. The typical variation in boundary position is ca 500 nm, consistent with the positional stability of the micropipettes obtained in Fig. 7.

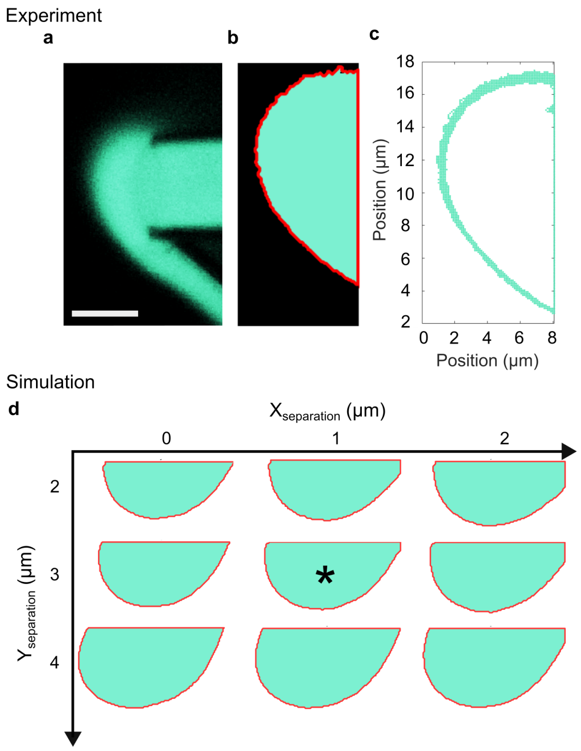

Figure 8: Stability of the flow Envelope. **a**, Confocal fluorescence image of a fluorescein flow envelope, established with $Q_{\mathrm{inj}} = 0.15\,\mathrm{nl\,s^{-1}}$ and $Q_{\mathrm{ratio}} = 0.2$ and imaged with an acquisition time of 55 ms. Scale bar is 5 μm. **b**, Mapped envelope boundary on a binarized (cutoff threshold = 5%) image. **c**, Composite image showing the variability of the envelope boundary extracted from 50 imaging frames. **d**, Numerical simulation of the brush geometry for a set of separation values in x- and y-dimension. Asterisk marks the envelope from the initial assumed position.

In tandem with the experimental observation of the envelope variability under normal operation, we also performed numerical simulation to explore how larger (i.e. $1\,\mu$m) variations in the inter-micropipette position would affect the envelope geometry (and hence boundary). Reflecting the experimental conditions of Fig. 8a-c, we also assume $Q_{\text{inj}} = 0.15\,\text{nl}\,\text{s}^{-1}$ and $Q_{\text{ratio}} = 0.2$ in our modeling of the micropipette-pair, shown in Fig. 8d, marked with asterisk. As in Fig. 8b, we present the envelope and boundary using a simplified threshold depiction of the true envelope. We consider an initial intended arrangement of the micropipettes (marked with an asterisk in Fig. 8d) comprising a $x_{\text{separation}} = 1\,\mu$m and $y_{\text{separation}} = 3\,\mu$m, and varied each parameter by $\pm 1\,\mu$m - corresponding to a relatively large drift/misalignment in intended position. Inspection of Fig. 8d finds no significant alteration to the flow envelope.

1.7.3 Reproducible Labeling

To characterize the consistency of labeling performance from event to event, we considered the number of beads that are deposited from a series of consecutive labeling events. We chose 200 nm fluorescent beads as our reagent of choice and functionalized a glass coverslip with Poly-L-Lysine to provide an attractive surface upon which the beads could readily adhere. The micropipette-pair was positioned close to the coverslip such that upon flow actuation, bead deposition begun immediately. The flow was ran equally for 10 s per patch before flow was ceased and the micropipettes moved to the next bare patch of coverslip.

The results of sequentially labeling the coverslip for 10 patches is shown in Fig. 9 for two envelope sizes; achieved by setting $Q_{\text{asp}} = 1.58\,\text{nl}\,\text{s}^{-1}$ and $Q_{\text{inj}} = 0.06\,\text{nl}\,\text{s}^{-1}$ (Fig. 9a) and $Q_{\text{inj}} = 0.23\,\text{nl}\,\text{s}^{-1}$ (Fig. 9b). We compute the variation in bead count to be $N = 21 \pm 4$ for the smaller envelope of Fig. 9a and $N = 128 \pm 9$ for the larger envelope of Fig. 9b.

As a second demonstration to characterize our labeling performance, this time considering a small molecule label, we performed CTxB-AF488 μkiss labeling on a supported lipid bilayer containing GM1 as previously performed in Fig. 2 of the main report. We performed 10 back-to-back μkisses for a kiss-time of 2 s with a $1\,\text{mg}\,\text{ml}^{-1}$ CTxB-AF488 solution. A montage of the labeled regions is shown in Fig. 9c,i-x for $Q_{\text{inj}} = 0.07\,\text{nl}\,\text{s}^{-1}$ and $Q_{\text{asp}} = 2.54\,\text{nl}\,\text{s}^{-1}$. The area of the labeled region, across all 10 samples is on average $65.6 \pm 7.3\,\mu\text{m}^2$, corresponding to a standard deviation of 11.3%.

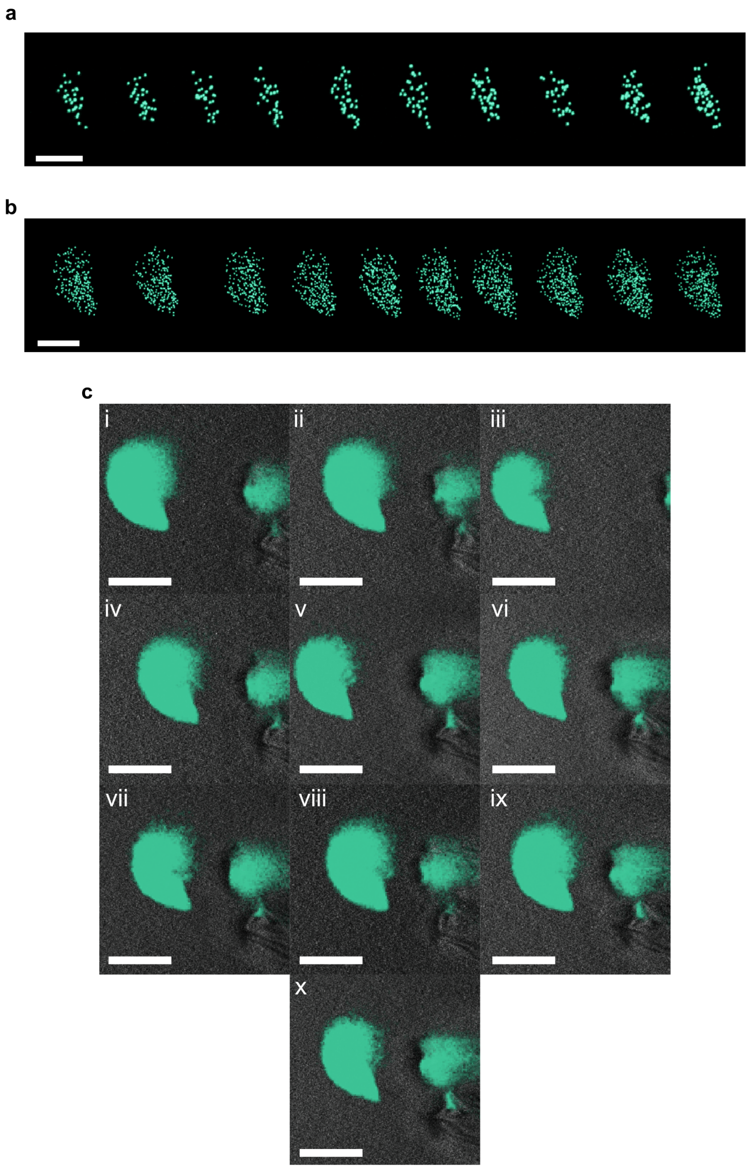

Figure 9: Reproducible deposition of beads via μkiss. Reproducible bead deposition on a substrate. **a**,**b** 10 patches of 200 nm fluorescent carboxylate beads deposited on a Poly-L-Lysine-functionalized coverslip with $Q_{\mathrm{inj}} = 0.06\,\mathrm{nl\,s^{-1}}$ and $Q_{\mathrm{ratio}} = 0.04$ (**a**), and $Q_{\mathrm{inj}} = 0.23\,\mathrm{nl\,s^{-1}}$ and $Q_{\mathrm{ratio}} = 0.15$ (**b**), respectively. The number of beads deposited is on average $N = 21 \pm 4$ (**a**) and $N = 128 \pm 9$ (**b**). Scale bar is 10 μm in (**a**) and (**b**). **c**,**i-x** A montage of composite images showing 10 back-to-back μkiss labelings of a SLB containing GM1 with an envelope of CTxB-AF488. The grayscale component shows a confocal iSCAT image and the green component shows the fluorescent CTxB-AF488 label. Scale bar is 5 μm.

2 μkiss Envelope Velocimetry

To determine the velocity of fluid flow within the brush envelope, gold nanoparticles are used as tracer particles and high-speed tracking of their positions is performed as they transit through the flowing envelope.

Gold nanoparticles with diameter 80 nm (BBI Solutions EM.GC80/7) were used at a concentration of 1.5×10^9 particles ml^{-1} (diluted 1:5 in Milli-Q water). A $1\,\mathrm{mg\,ml}^{-1}$ stock solution of fluorescein was prepared (sodium salt, Merck, 518-47-8) and was added in a 1:100 dilution for visualizing the flow envelope through fluorescence. The brush envelope established for the particle velocimetry measurement is shown in Fig. 10a for $Q_{\mathrm{inj}} = 0.11\,\mathrm{nl\,s}^{-1}$ and $Q_{\mathrm{ratio}} = 0.27$.

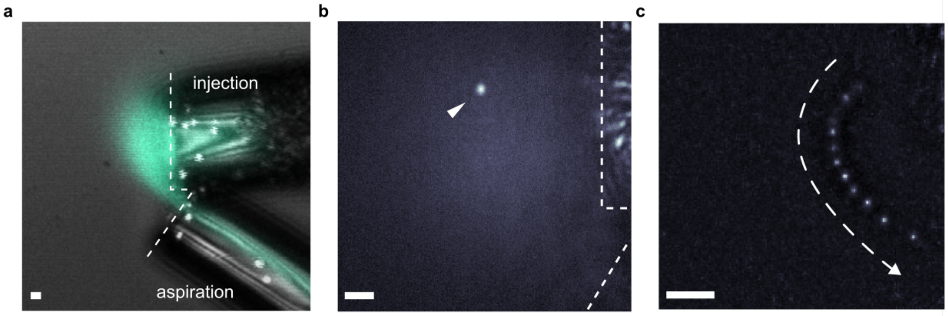

Figure 10: Velocimetry of flow envelope through tracking of nanoparticle tracers a, dual confocal iSCAT (gray scale) and confocal fluorescence (green) image showing the positioning of the μkiss apertures and the resulting fluorescently-labeled flow envelope. Dashed lines guide the eye to the micropipette apertures. Scale bar is $1\,\mu$m. **b**, Raw widefield iSCAT image from the the field of view of (**a**) with a single nanoparticle particle mid-transit. Dashed lines guide the eye to the micropipette apertures, as shown in (**a**). Scale bar is $1\,\mu$m. **c**, Composite RVT image from a particle tracked across consecutive frames in time. The dashed line guides the eye to the transit path of the particle. Scale bar is $1\,\mu$m.

Figure 10b shows a single widefield iSCAT imaging frame wherein a single gold nanoparticle is mid-transit. Widefield iSCAT imaging and particle tracking is performed according to Taylor *et al.*[4]. In this work, the exposure time is $10\,\mu$s and the framerate 10,000 fps. Each imaging frame containing a nanoparticle that is to be tracked is firstly processed by removal of static background features through subtraction of the temporal-median background. Each frame is then converted using the Radial Variance Transform (RVT), with filter radii corresponding to 3, 5 and 7 pixels, to remove ring-features of the point-spread function (PSF)[5]. Figure 10c presents a composite image of the RVT-transformed PSF for consecutive frames for a single gold nanoparticle that is tracked. To each transformed PSF, such as those in Fig. 10c, a two-dimensional Gaussian function is fitted to determine the particle center. By computing the displacement of the particle between pairs of frames, the instantaneous pair-wise flow velocity is calculated.

3 Characterization of the Flow Envelope

3.1 Parameterizing Envelope Extent

To characterize the physical size of the flow envelope, we introduce the extent parameter, L_e, which represents the length by which the envelope protrudes out of the injection pipette and into the open fluidic medium, shown in Fig. 11.

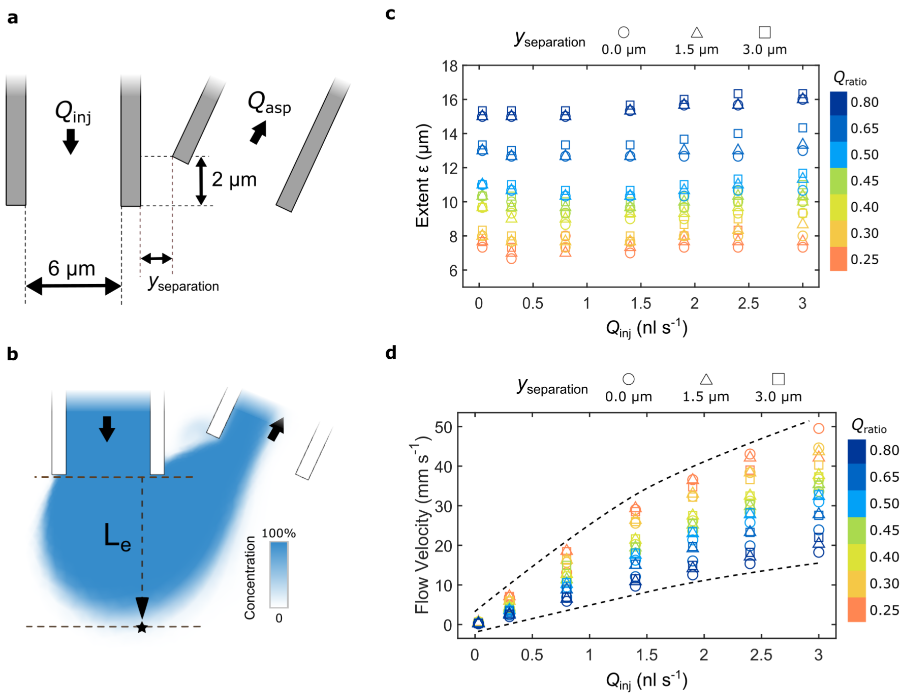

Figure 11: Numerical simulation of the μkiss envelope extent and associated flow velocity. **a**, Schematic of μkiss apertures geometry used for numerical simulation. Arrows mark the direction of fluid flow. **b**, Computed distribution of reagent material within the flow envelope system for $y_{separation} = 1.5\,\mu m$. The size parameter - 'extent', L_e, is highlighted. An asterisk-marker guides the eye to the point on the envelope boundary where L_e terminates. **c**, Computed extents for flow envelopes as a function of Q_{inj}, Q_{ratio} and micropipette separations. **d**, Computed flow velocities at extent apex (asterisk marker) as a function of the full parameter set for Q_{inj}, Q_{ratio} and micropipette separations.

We numerically model the μkiss envelope using the arrangement shown in Fig. 11a,b, for a range of Q_{inj}, Q_{asp} and also $y_{separation}$. For clarity, we express Q_{asp} through its value relative to Q_{inj}, that is: $Q_{ratio} = Q_{inj}/Q_{asp}$. The results of numerical simulation is summarized in Fig. 11c. One sees the extent of the envelope is a balance between the injection flow rate Q_{inj} and aspiration flow rate Q_{asp}; independent of micropipette positions, a higher Q_{inj}, relative to Q_{asp}, promotes a larger L_e. Alternatively, one may interpret Fig. 11c to indicate that larger Q_{asp} act to curtail L_e for all scenarios to render a smaller and compact envelope.

It is also instructive to inspect the flow velocity v of the envelope at the boundary point defined by $\mathrm{L_e}$ (marked by a star in Fig. 11b). In Fig. 11d, the flow velocity at L_e, denoted v_e, is presented.

3.2 Reynolds Number at the Envelope Boundary

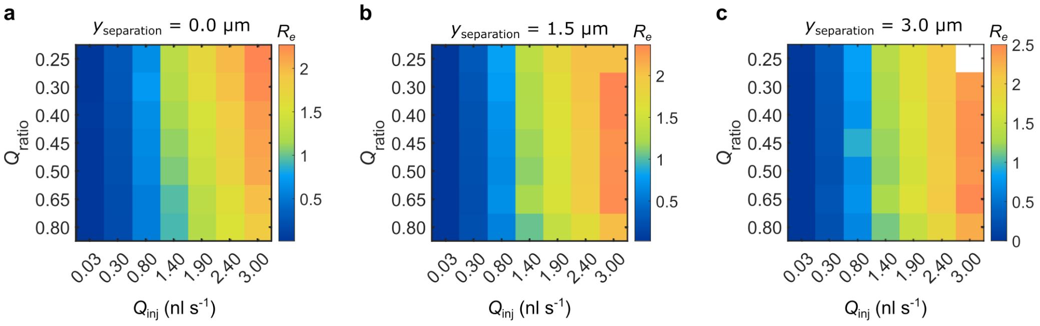

Figure 12: Reynolds numbers computed for the μkiss envelope. Mean Reynolds numbers R_e along the flow envelope boundary for various Q_inj and Q_ratio for $y_\mathrm{separation} = 0.0\,\mu$m (**a**), $1.5\,\mu$m (**b**) and $3.0\,\mu$m (**c**).

The Reynolds number provides an indication whether a fluid flow in a particular context is laminar and regular, or turbulent, and can be expressed via:

$$R_\mathrm{e} = \frac{\rho v L_\mathrm{R}}{\mu} \tag{2}$$

where μ stands for viscosity and ρ signifies the density, in this case of water with values of 0.9544 mPa s and 997 Kg m^{-3}, respectively. The parameter L_R denotes the path along which flow occurs, and we may approximate $L_\mathrm{R} = \pi\,\mathrm{L_e}$ and take the flow velocity v as the flow velocity at maximum extent (i.e. v_e occurring at $\mathrm{L_e}$). The result of the calculation for R_e for various Q_inj, Q_ratio and $y_\mathrm{separation}$ is presented in Fig. 12. Across our system, $R_\mathrm{e} = 0.006 - 2.5$ and therefore the assumption of laminar flow is supported.

3.3 Péclet Number at the Envelope Boundary

The Péclet number is a measure of the convective versus diffusive transport, and helps one evaluate whether one can achieve strong confinement of a dilute species - that is, a sharp boundary between the reagents of the confined envelope and the open fluidic environment, which should otherwise be free of the reagents. In a pragmatic sense, the Péclet number identifies if the flow velocity is 'too slow', and is permitting sufficient time for the reagents to diffuse out of the regular directed path of the laminar flow jet. Such an effect manifests as a broadening of the envelope boundary, depicted in Fig. 13 for two such cases where the injection rate is the same ($Q_\mathrm{inj} = 0.03\,\mathrm{nl\,s^{-1}}$) but the Q_asp varies. In Fig. 13a,b - where $Q_\mathrm{ratio} = 0.80$, diffusion-led broadening is larger. Inspecting the numbered markers as one progresses around the envelope reveals a widening of the envelope boundary (Fig. 13a), which is a result of the low flow velocities in this region (Fig. 13b). We define the edge of the boundary to be the point at

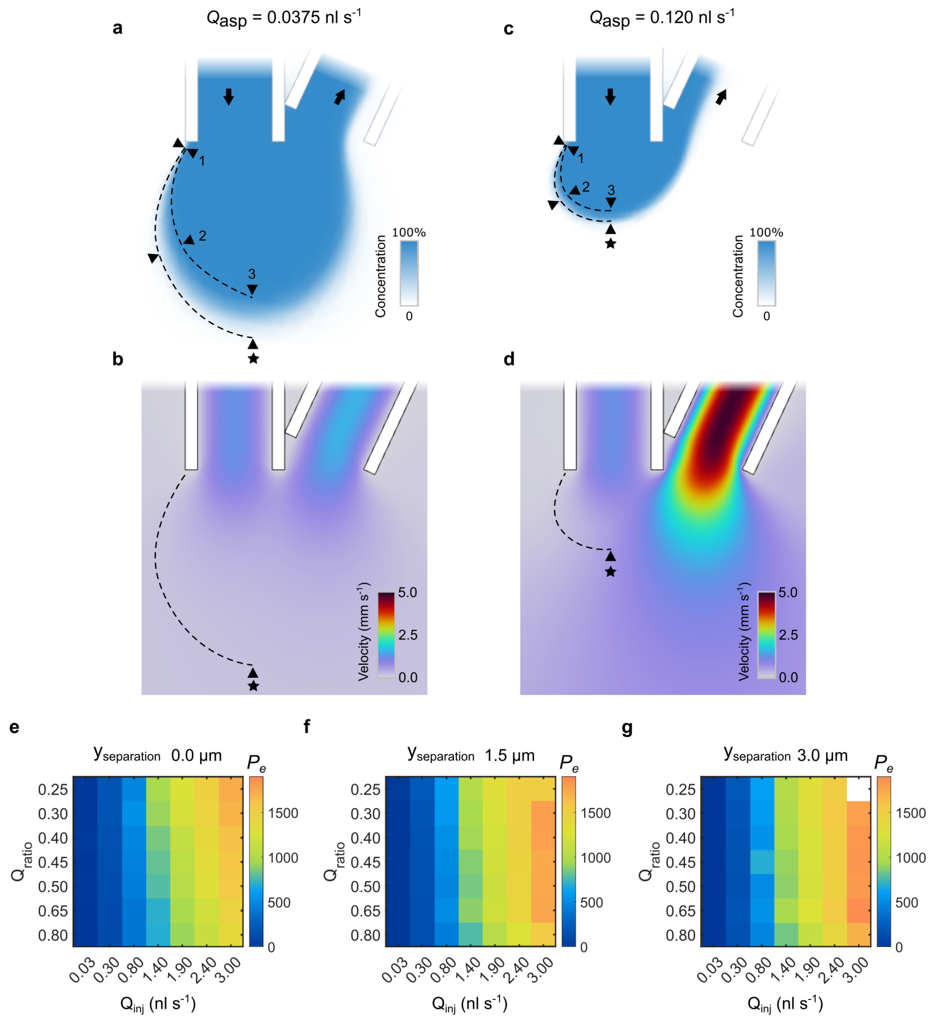

Figure 13: Diffusion-led broadening of the flow envelope and Péclet number. **a**, Simulated steady-state distribution of reagent (blue) within a flow envelope with $Q_{\rm inj} = 0.03\,{\rm nl\,s^{-1}}$ and $Q_{\rm asp} = 0.0375\,{\rm nl\,s^{-1}}$ **a**, **b**, and the corresponding flow velocity field $\boldsymbol{v}$ (**b**). Star marker guides eye to corresponding feature points. **c**, a contrasting scenario to (**a**), with here a larger $Q_{\rm asp} = 0.12\,{\rm nl\,s^{-1}}$. and $Q_{\rm inj} = 0.03\,{\rm nl\,s^{-1}}$. **d**, The corresponding flow velocity field from (**c**). Star marker guides eye to corresponding feature points. **e**, Péclet number P_e along the flow envelope boundary for various $Q_{\rm inj}$ and $Q_{\rm ratio}$ for (**e**), $y_{\rm separation} = 0.0\,\mu$m, **f**, $1.5\,\mu$m and **g** $3.0\,\mu$m .

which the concentration of the envelope falls to 1%. The star marker acts as a guide to the eye. By increasing $Q_{\rm asp}$, shown in Fig. 13c,d, the envelope boundary remains crisp (Fig. 13c) as the flow velocity in this region is now larger (Fig. 13d). In both cases, shown in Fig. 13, the reagents remain confined to the envelope, only their distribution at the envelope is affected. In addition, past the point of maximum extent, the boundary becomes more confined owing to the higher flow velocities associated with the aspiration micropipette. For this reason, the envelope at the point of ϵ - shown by the star marker in Fig. 13, is the point where broadening is often largest.

The Péclet number (P_{e}) parameterizes the strength of diffusion-led broadening of convective flow, and is given by:

$$P_{\mathrm{e}} = \frac{vL_{\mathrm{R}}}{D} \tag{3}$$

where D is the diffusion constant of the reagent, here $D = 1.34\,\mu\mathrm{m}^2\mathrm{s}^{-1}$, and we again take the relevant fluid velocity v as that occurring at the peak extent. The result of our numerical modeling of P_{e} is summarized in Fig. 13e-g for various Q_{inj}, Q_{ratio} and $y_{\mathrm{separation}}$. For our system, we find $P_{\mathrm{e}} \approx \times 10^2$, that is, $P_{\mathrm{e}} >> 1$, and hence the flow-based confinement dominates over diffusive processes.

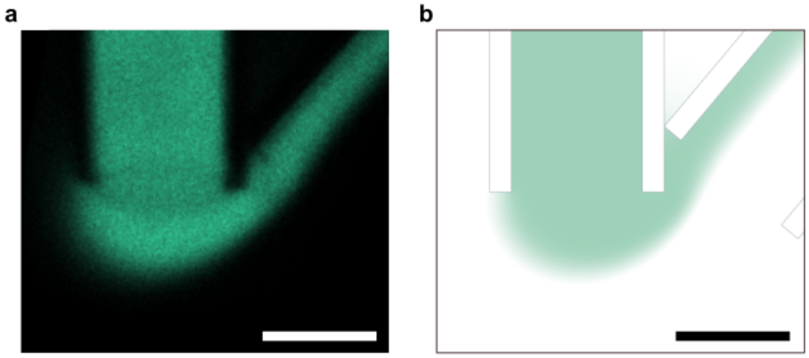

Figure 14: Hydrodynamic flow confinement of a small-molecule reagent (fluorescein). **a**, Confocal fluorescence image of a fluorescein ($1\,\mathrm{mg\,ml}^{-1}$) flow envelope established with $Q_{\mathrm{inj}} = 0.16\,\mathrm{nl\,s}^{-1}$ and $Q_{\mathrm{ratio}} = 0.3$. **b**, Numerical simulation of the flow envelope with corresponding geometry created with Q_{inj} and Q_{ratio}. Scale bar is $5\,\mu$m for both.

To demonstrate that our flow-based confinement is sufficient for most labeling applications, in Fig. 14 we show flow confinement of a small-molecule reagent. We chose fluorescein which has a low mass of 332 Da and a high diffusivity of $D = 435\,\mu\mathrm{m}^2\,\mathrm{s}^{-1}$. As shown in Fig. 14a, using standard flowrates ($Q_{\mathrm{inj}} = 0.16\,\mathrm{nl\,s}^{-1}$ and $Q_{\mathrm{ratio}} = 0.3$), the fluorescein reagent is able to be confined within the flow envelope. To further verify our observations we performed numerical modeling of the scenario shown in Fig. 14a, presented in Fig. 14b, which also demonstrates flow confinement of the small-molecule reagent.

4 Cell Viability Under μkiss Operation

To assess the viability of cells subject to μkiss brush delivery, the LIVE/DEAD assay (Invitrogen, R37601) is used. The LIVE signal indicates metabolic activity and hence cell health, while DEAD signal arises due to a loss of cell membrane integrity and cell death. To firstly verify that we are able to correctly identify both the LIVE and DEAD signals from a sample transitioning from a healthy to distressed state, we perform μkiss brush delivery of an insulting reagent to a select single cell. We chose the insulting reagent to be a 1% Triton X100 membrane detergent solution, which has an immediately lethal effect on the live cell.

Figure 15a presents a dual-channel image of a healthy cell culture labeled with the LIVE/DEAD stain. μkiss brush delivery is performed on the cell marked with the white arrow, additionally depicted in Figure 15b. Post insult, the targeted cell shows a complete absence of the previous LIVE signal, instead displaying a strong DEAD fluorescence signal - shown in Fig. 15c. A time trace of the LIVE and DEAD signal, integrated across the entire cell, for the marked cell is plotted in Fig. 15d wherein the concomitant transition upon insult is observed.

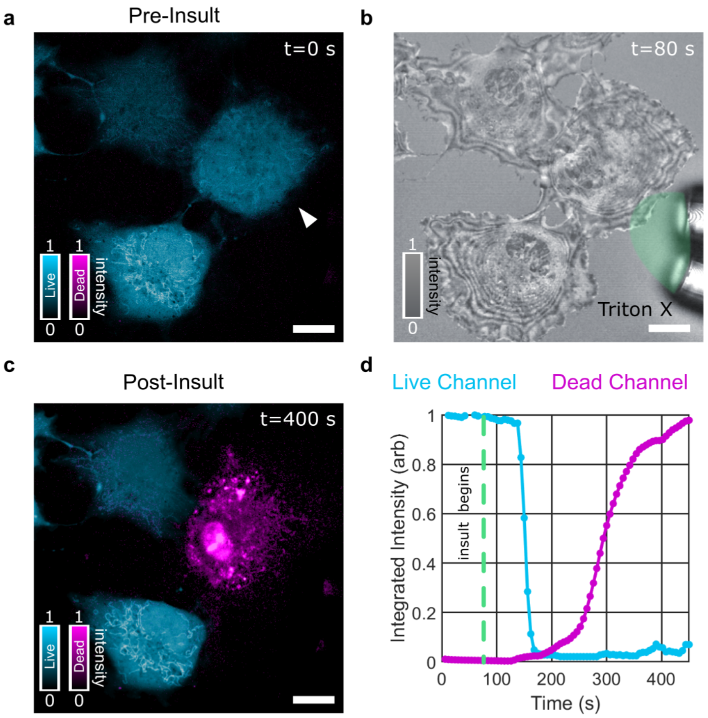

Figure 15: Validating LIVE/DEAD assay on a single cell. **a**, Dual-channel confocal fluorescence image of a cell culture treated with both the LIVE and DEAD label, prior to insult. Arrowhead marks intended target cell. **b**, iSCAT image corresponding to (**a**), showing μkiss envelope position and overlay of Triton X100 flow envelope (green). **c**, Dual-channel confocal fluorescence image of the region shown in (**a**) following Triton X100 delivery to the marked cell of (**a**). Result is indicative of a series of independent measurements of size $n = 3$. **d**, Integrated intensity from the marked cell showing the LIVE and DEAD signals as a function of time. Green dashed line denotes start point of Triton X100 insult. Scale bars 5 μm throughout figure.

To now assess the effect of μkiss brush delivery alone on cell health, we perform a similar measurement wherein a benign reagent is applied to the cell, shown in Fig. 16. We chose cell culture medium as the nominal reagent and supplement it with a small quantity of Transferrin-AF647 for visualizing the flow envelope. Figures 16a-c present the target healthy cell which displays an exclusive LIVE signal. Following continuous μkiss brush delivery for 30 minutes, shown in Fig. 16d-f, inspection reveals an absence of DEAD signal, nor a change in the LIVE signal nor an observable change in the brightfield-based iSCAT image. The flow rate here represents the largest used throughout this work ($Q_{\mathrm{inj}} = 0.55\,\mathrm{nl\,s^{-1}}$ and $Q_{\mathrm{asp}} = 1.60\,\mathrm{nl\,s^{-1}}$). These results confirm that extended and direct operation of the μkiss brush does not affect viability of the targeted cell.

To further validate the viability of μkiss labeling, we repeated benign delivery on a greater ensemble of cells ($n = 10$) for an extended period of 30 min. In order to quantify the true positive DEAD signal, to which our benign delivery is referenced, we also induced cell death by μkiss delivery of a strongly toxic agent (1% Triton X100), shown in yellow of Fig. 16g. The results of our extended ensemble labeling is summarized in Fig. 16h, wherein it is evident that μkiss labeling does not promote any activation of the DEAD signal, affirming the viability of the approach across a greater cellular ensemble.

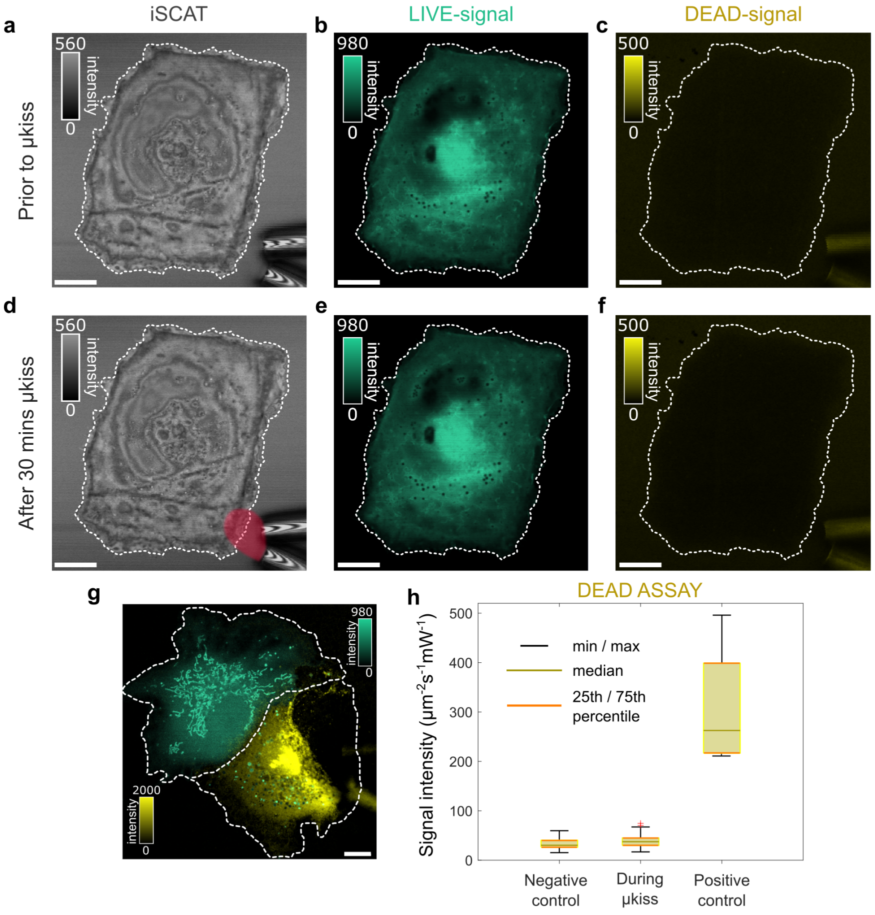

Figure 16: Viability of μkiss brush delivery. **a-c**, Separate image channels of a chosen target cell, prior to μkiss brush operation, viewed as iSCAT (brightfield, (**a**)) as well as the LIVE (**b**) and DEAD (**c**) fluorescence channels. **d-f**, Corresponding image channels to (**a**)-(**c**), following 30 min of continuous μkiss brushing wherein cell culture medium is locally applied to the region marked by the red overlay. **g**, Confocal fluorescence image of the LIVE/DEAD-signals of a pair of adjacent cells, with the target cell being exposed to a lethal μkiss. Results illustrative of a series of $n = 4$ measurements. **h**, Box plot showing the recorded DEAD-signal of the negative control (before μkiss), during 30 min of continuous benign μkiss and the positive control (lethal μkiss). The negative control comprises a sample size of $n = 5$, with the distribution having a median and standard deviation 30.2 $\pm$ 10.7 $\mu m^{-2}s^{-1}mW^{-1}$, 25th and 75th percentiles of 26.2 $\mu m^{-2}s^{-1}mW^{-1}$ and 40.4 $\mu m^{-2}s^{-1}mW^{-1}$ respectively, and a minimum and maximum of 15.2 $\mu m^{-2}s^{-1}mW^{-1}$ and 59.7 $\mu m^{-2}s^{-1}mW^{-1}$, respectively. The 'During μkiss' measurement comprises a sample size of $n = 10$, with the distribution having a median and standard deviation 37.4 $\pm$ 10.4 $\mu m^{-2}s^{-1}mW^{-1}$, 25th and 75th percentiles of 29.8 $\mu m^{-2}s^{-1}mW^{-1}$ and 45.1 $\mu m^{-2}s^{-1}mW^{-1}$ respectively, and a minimum and maximum of 16.8 $\mu m^{-2}s^{-1}mW^{-1}$ and 74.7 $\mu m^{-2}s^{-1}mW^{-1}$, respectively. The positive control comprises a sample size of $n = 4$, with the distribution having a median and standard deviation 262.5 $\pm$ 114.7 $\mu m^{-2}s^{-1}mW^{-1}$, 25th and 75th percentiles of 216.5 $\mu m^{-2}s^{-1}mW^{-1}$ and 398.9 $\mu m^{-2}s^{-1}mW^{-1}$ respectively, and a minimum and maximum of 210.0 $\mu m^{-2}s^{-1}mW^{-1}$ and 496.0 $\mu m^{-2}s^{-1}mW^{-1}$, respectively. Scale bar is 5 μm throughout.

5 Temporal Response of μkiss Brushing

The temporal response of μkiss brushing is defined as the amount of time that is required for the brush envelope to establish itself, following actuation of flow. To characterize the response time, the development of the brush as a function of time is recorded with the envelope extent used to parameterize the envelope. We define the edge of the boundary to be the point at which the concentration of the envelope falls to 1%.

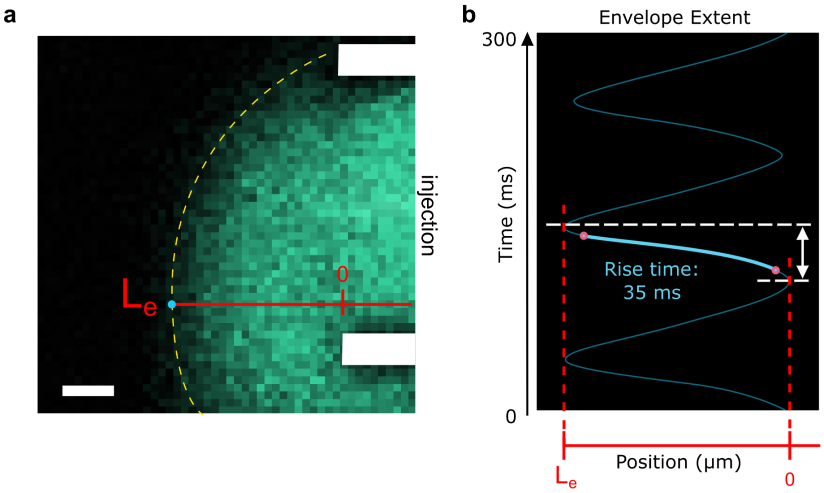

Figure 17: Temporal response of the μkiss envelope a, Confocal fluorescence image of an established, fluorescently-labeled flow envelope. The dashed line guides the eye to the envelope boundary (threshold = 1% of the fluorescence intensity). The solid red line marks the path along which the maximum envelope extent ($\mathrm{L_e}$) is extracted. The white regions guide the eye to the walls of the injecting micropipette. Scale bar is 1 μm. **b**, A time-series of line scans through the maximum extent region of the envelope was recorded (Scan time = 229 μs), with alternating cycles of $Q_{\mathrm{inj}} = 0.0\,\mathrm{nl\,s^{-1}}$ and $Q_{\mathrm{inj}} = 0.076\,\mathrm{nl\,s^{-1}}$. $\mathrm{L_e}$ (with $Q_{\mathrm{ratio}} = 0.13$) plotted as a function of time, revealing a rise time of 35 ± 2.5 ms.

Figure 17a presents a confocal fluorescence image showing a close-up view of a brush envelope (visualized by inclusion of a fluorescent dye AF488), with the path along which the extent is measured. With a line-imaging time of 229 μs, the intensity of the signal along the path marked $\mathrm{L_e}$ in Fig. 17a is recorded, and summarized in Fig. 17b for $Q_{\mathrm{inj}} = 0.076\,\mathrm{nl\,s^{-1}}$ and $Q_{\mathrm{asp}} = 3.20\,\mathrm{nl\,s^{-1}}$ for each point in time of several actuation cycles of Q_{inj}. The rise time - the time for the envelope to develop from 10% to 90% of its maximum extent - is here found to be 35 ± 2.5 ms.

6 μkiss & FRAP: Analysis & Modeling

To determine the diffusion constant of AF488-CTxB-labeled GM1 via FRAP microscopy, we follow the analysis procedure outlined in Ref. 6. For FRAP performed with a confocal bleach spot, the diffusion constant D_{FRAP} can be found via:

$$D_{\mathrm{confocal}} = \frac{r_e^2 + r_n^2}{8\,\tau_{1/2}} \tag{4}$$

where r_n is the nominal radius of the bleach spot (from assumed ideal values), r_e is the 'experimental' radius of the bleach spot and is directly measured, and finally $\tau_{1/2}$ is the half-life of the recovering bleached intensity. An example of a FRAP measurement is shown in Fig. 18a. To perform the analysis, on each imaged bleached frame a two-dimensional Gaussian function is fitted to the intensity distribution. From this fit, the peak intensity I of the bleached spot can be extracted, shown in Fig. 18b, for all times during bleaching recovery (yielding $I(t)$). To compute the half-life for bleach recovery, an exponential function of the form $I(t) = a\,e^{(-b\,t)} + c$ is fitted to the extracted amplitudes, where a, b and c are variables to be fitted. The half-life of $I(t)$ is then $\tau_{1/2} = log(2)\,b^{-1}$. The nominal radius of the confocal bleaching spot can be calculated via $r_n = \lambda/(4\mathrm{NA})$, where λ is the wavelength (here 475 nm) and NA is the numerical aperture (here, 1.4). The experimental radius can be extracted via the two-dimensional Gaussian function fitted to the first post-bleached frame (such as in Fig. 18a). With these computed, the diffusion D_{FRAP} constant can be calculated.

The framework used to calculate the diffusion constant for a FRAP measurement can also be applied to a μkiss brush labeling measurement, illustrated in Fig. 18c. To each imaged frame of the CTxB-AF488:GM1 complex, a two-dimensional Gaussian function can be fitted in order to extract the peak amplitude of the labeled spot across time ($I(t)$) - shown in Fig. 18d. Similar to the FRAP analysis, to the $I(t)_{\mu\mathrm{kiss}}$ one can also fit an exponential function to the distribution to yield the half-life of the diffusion-led amplitude decay. As a nominal radius of the spot size is not applicable, we assume $r_n = r_e$, where r_e is extracted from a two-dimensional Gaussian function fitted to the first labeled frame of the set. Following this, the diffusion constant $D_{\mu\mathrm{kiss}}$ can be calculated.

To validate our analysis approach for extracting a diffusion constant from our μkiss measurement, we perform numerical modeling of an analogous system. We use the FRAP modeling framework of Ref. 7, and invert the amplitude so-as to mimic the intensity distribution characteristic of μkiss. Fig. 18e presents a simulated image sequence of diffusion-led broadening of an intensity distribution. We adopt a pixel size similar to that of our imaging confocal microscope pixel size and chose a time resolution of 0.1 s. We assume an initially circular intensity distribution, with radius 1 μm, and with soft edges (convolution with a Gaussian) and add noise to mimic a signal-to-noise ratio (SNR) of 10, as encountered in experiment. Finally, we assert the designed diffusion constant to be $D^*_{\mu\mathrm{kiss}} = 0.05\,\mu\mathrm{m}^2\mathrm{s}^{-1}$. Repeating the analysis procedure used for experimental μkiss, outlined above, we compute the model diffusion constant $D^{\mathrm{model}}_{\mu\mathrm{kiss}}$ for n = 25 runs, with a histogram summarizing the distribution in Fig. 18f.

7 Sub-cellular Delivery Targeting the Microtubule Network

To verify that the observed sub-cellular insult response results from the chemical function of demecolcine, and is not an artefact of the means by which it is delivered, i.e. from μkiss brush delivery, we performed a similar measurement

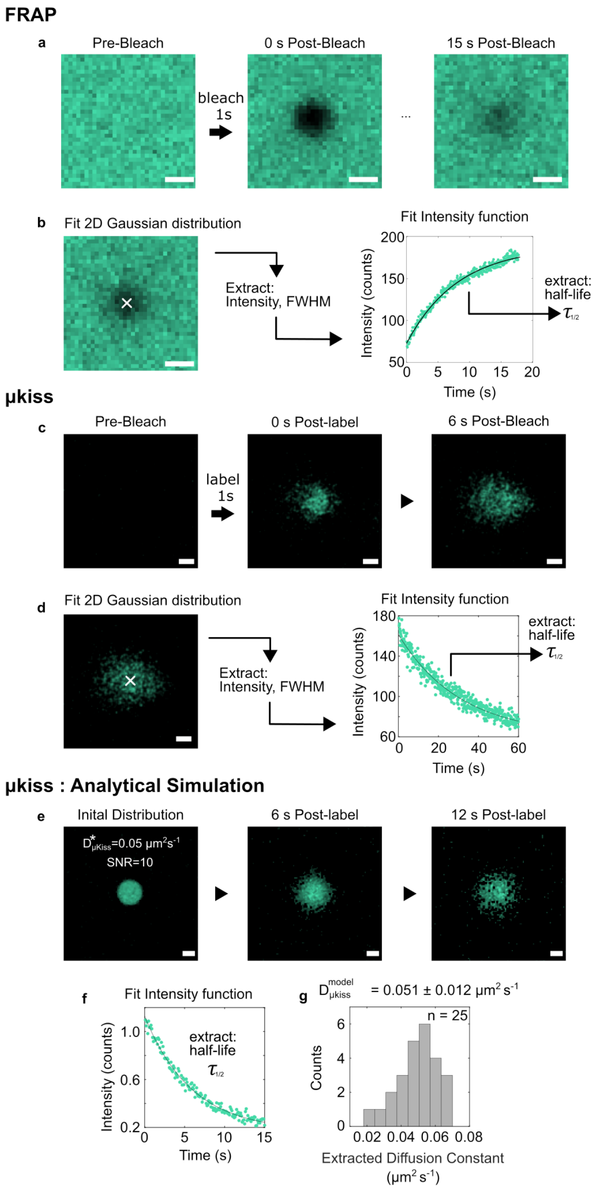

Figure 18: Determination of GM1 diffusion constant for FRAP and μkiss a, Confocal fluorescence image sequence showing FRAP bleach and recovery of the CTxB-AF488:GM1 SLB. **b**, Schematic showing process of parameter extraction from (**a**), and characteristic plot of extracted bleached-signal intensity during recovery time from which $\tau_{1/2}$ is fitted. Scale bars in (**a**) and (**b**) 1 μm. **b**, Confocal fluorescence image sequence showing μkiss brush labeling and diffusion of CTxB-AF488 labeled GM1 SLB. **d**, Schematic showing process of parameter extraction from (**c**), and characteristic plot of extracted signal region over time, from which $\tau_{1/2}$ is fitted. Scale bars in (**c**) and (**d**) 1 μm. **e**, Numerical simulation of a μkiss labeling measurement, where mobile species has diffusion constant $D^*_{\mu\text{kiss}}$ and each image frame has a SNR of 10. **f**, Extracted peak signal intensity of the 'labeled' region, following fit of a two-dimensional Gaussian function. **g**, Histogram of extracted diffusion constant of mobile species for $n = 25$ repeated runs.

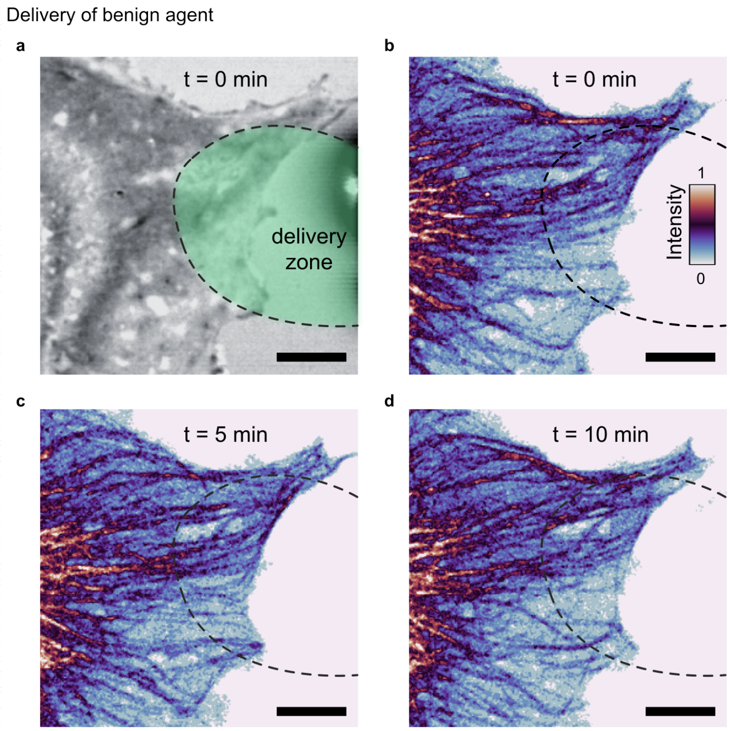

Figure 19: Delivery of a non-insulting reagent does not perturb the microtubuli network. **a**, iSCAT image of a target cell, to which Leibovitz's medium will be delivered. The region of local delivery is marked in green for reference. **b**, Confocal fluorescence image of the region in (**a**), revealing the pmEGFP-labeled microtubule network prior to Leibovitz's delivery. Leibovitz's medium is applied to the marked region continuously and imaged after 5 min, (**c**), and 10 min, (**d**). Scale bar is 5 μm throughout.

wherein we deliver warmed Leibovitz's cell culture medium as a benign reagent. Figure 19a shows an iSCAT image of the sub-cellular region of interest, with the outline of the targeting zone marked for reference.

A cell culture is transfected to express an α-tubulin-pmEGFP fusion protein to visualize the microtubule network, shown in Fig. 19b for the same region. μkiss delivery of warmed Leibovitz's was performed continuously for 10 min at the same experimental conditions of demecolcine application in Fig. 3 of the main report ($Q_{\mathrm{inj}} = 0.23\,\mathrm{nl\,s^{-1}}$ and $Q_{\mathrm{asp}} = 1.59\,\mathrm{nl\,s^{-1}}$). Confocal fluorescence images following the microtubule network at 5 min (Fig. 19c) and 10 min (Fig. 19d) of continuous application reveal an absence of network disruption as it was observed under demecolcine application.

The changes in length of the microtubules affected by demecolcine insult, presented in Fig. 3 of the main report, is found as follows: Fig. 20a presents a confocal fluorescence image of the EGFP-labeled microtubule network from Fig. 3 (main report), prior to demecolcine insult. Scale bar is 5 μm. Intensity scale used throughout entire figure. The five microtubules under inspection are highlighted in Fig. 20, each with a unique color and a star marker denoting the tip location prior to retraction. The result of demecolcine insult leads to retraction of each microtubule, with the retracting tip position marked along the respectively-colored trajectory. The arrow marker indicates the general

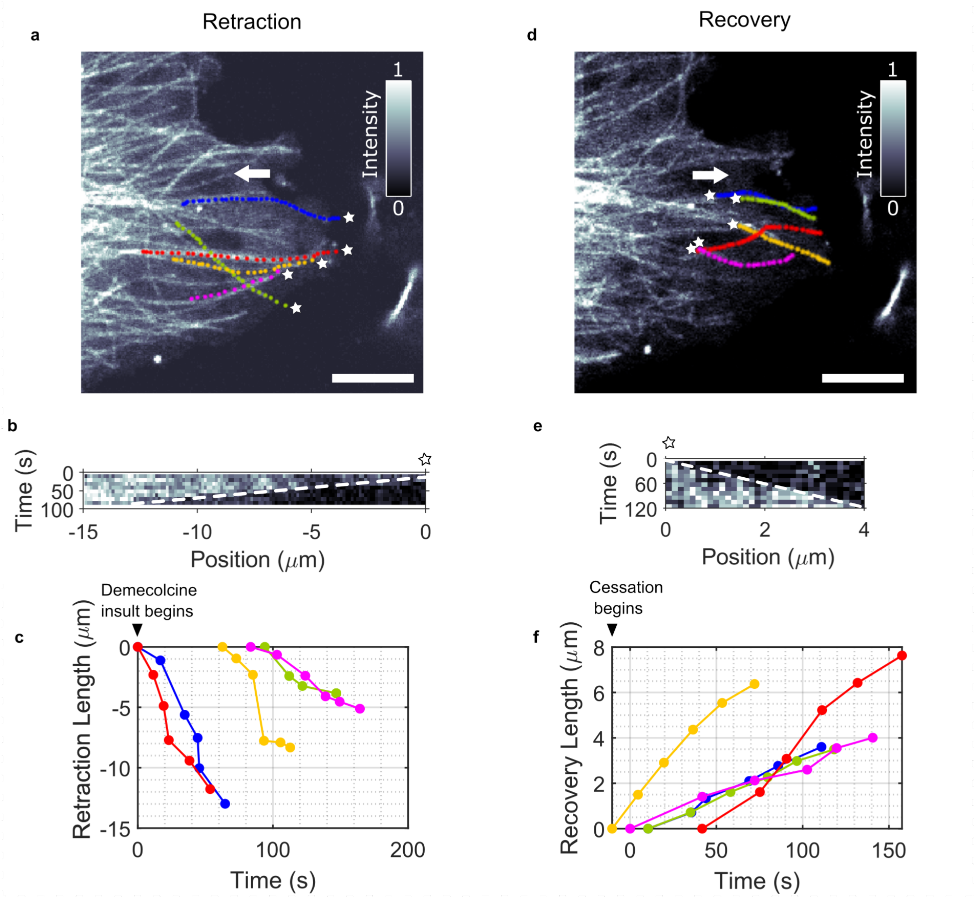

Figure 20: Local-scale retraction and recovery of the microtubule network in response to local demecolcine insult. **a**, Fluorescence image of a cell with the microtubule network labeled by overexpression of an α-tubulin-pmEGFP fusion protein. The positions of the tips from five retracting microtubules are plotted as a function of time, with star marker denoting the initial positions. Arrow marker indicates general retraction direction. Scale bar 5 μm. **b**, Kymograph of microtubule retraction for the path labeled blue in (**a**). Dashed line guides the eye to the tip position. **c**, The length of microtubule tip retraction for the five tips indicated in (**a**), as a function of time from point of demecolcine application. **d**, Fluorescence image of the region in (**a**), at the point of demecolcine insult cessation. Star markers denotes initial position of microtubule tips, and colored paths respectively indicate the advancing tip positions during regrowth (recovery). Arrow marker denote direction of regrowth. Scale bar 5 μm. **e**, Kymograph of microtubule recovery for the path labeled blue in (**d**). **f**, The length of microtubule tip recovery for the five tips indicated in (**f**), as a function of time from point of demecolcine insult cessation.

direction of retraction movement. Each trajectory point corresponds to a subsequent imaging frame of 10 s. Taking the trajectory of tip retraction, a kymograph of pmEGFP-microtubule intensity along this path can be constructed, presented in Fig. 20b for the blue trajectory in Fig. 20a. The star marker denotes again the initial tip position prior to insult. The dashed line in Fig. 20b guides the eye to the tip position during retraction. In Fig. 20c we present the full set of tip retractions along their respective retraction trajectory - written as 'Retraction Length' - as a function of time elapsed since demecolcine application. The length-change here is defined to be negative, to emphasize a net loss in length. One observes the start time of retraction occurs later for microtubules that are initially positioned further away from the μkiss envelope, suggesting indeed a concentration gradient of applied demecolcine within the cell interior.

Upon cessation of demecolcine delivery, 9 min after beginning, shortened microtubules are observed to grow longer and so return to lengths similar to their state prior to demecolcine insult. Figure 20d highlights five such microtubules that undergo a recovery in their length. In analogy to Fig. 20a, the star marker denotes initial tip position prior to recovery. The arrow marks general direction of recovery growth. Each colored data marker plots the tip position for each 10 s imaging frame. A kymograph of the pmEGFP-microtubule intensity along the regrowing trajectory for the tip marked in blue in Fig. 20a is presented in Fig. 20b, with the dashed line guiding the eye to the tip position. The lengths 'regrown' along each recovery trajectory - written as 'Recovery Length' for all five microtubules is plotted in Fig. 20f as a function of time, beginning from the point of demecolcine cessation.

8 Manders' Coefficient of Tf-AF647:Clathrin Labeling

In Fig. 4b of the main report, we present co-labeling of clathrin and TfR, the latter labeled by μkiss brush delivery of Tf-AF647. To quantify the degree of co-localization, we calculate Manders' coefficients m to find the percentage of total signal from one channel which overlaps with signal from the other. Figure 21a presents an iSCAT image of the cell region prior to labeling of Tf-AF647. The clathrin distribution within the marked region post-labeling of Tf-AF647 is shown in Fig. 21b, with the Tf-AF647 signal in the same region shown in Fig. 21c. Dashed line marks the extent of the μkiss brush labeling region. A composite merge of Figs. 21b,c is shown in Fig. 21d for reference.

Following the approach of Supplementary Ref. 8, we compute the coefficients. Here, one channel corresponds to the clathrin signal and the other to that of Tf-AF647. We compute the clathrin spatial overlap with Tf-AF647 by summing the intensity of every pixel with clathrin intensity whose corresponding Tf-AF647 pixel is above zero, and normalize to the total original clathrin signal. Doing so we compute $m = 142/295 = 0.62$. Similarly we find for Tf-AF647: $m = 129/183 = 0.71$.

9 Quantifying TfR Labeling

To estimate the number of TfR that we label with μkiss brush delivery of Tf-AF488, we need to calibrate the optical signal of Tf-AF488 to the number of Tf present, assuming 1:1 binding of Tf and TfR. To begin, we first quantify the ratio of AF488 to Tf in our labeling reagent solution. We use a NanoPhotometer (Implen GmbH) to measure the optical extinction of our $2\,\mathrm{mg\,ml^{-1}}$ Tf-AF488 solution, and find the concentration of AF488 to be four-fold higher than that of the Tf, giving a labeling ratio of 4:1. At a working Tf-AF488 concentration of $2\,\mathrm{mg\,ml^{-1}}$, Tf is present as $C_{\mathrm{Tf}} = 15055\,\mathrm{molecules}\,\mu\mathrm{m}^{-3}$ and AF488 as $C_{\mathrm{AF488}} = 60220\,\mathrm{molecules}\,\mu\mathrm{m}^{-3}$.

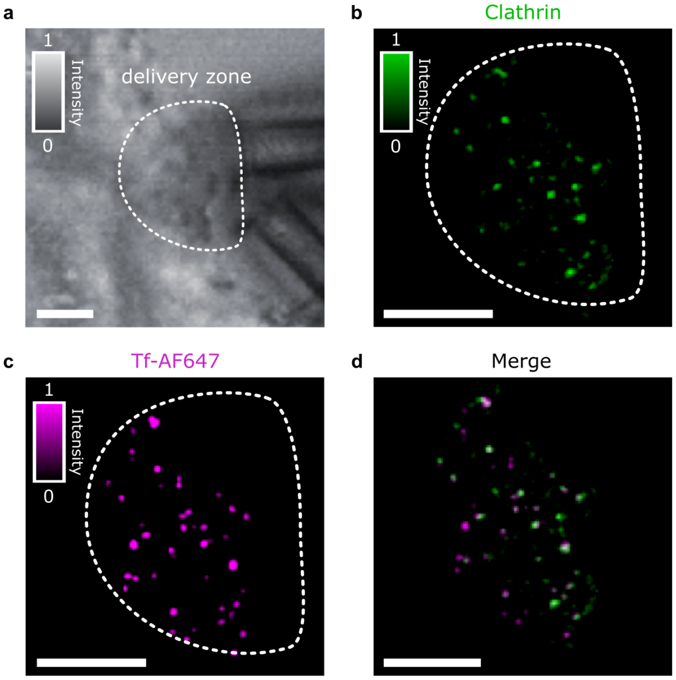

Figure 21: Tf-AF647 and clathrin distribution used for calculating Manders co-localization coefficient. **a**, iSCAT image of the target cell region, with dashed line marking the labeling area. **b**, Fluorescence image showing clathrin distribution within the dashed region from (**a**) at the point immediately following Tf-AF647 delivery to the dashed region. **c**, Fluorescence image showing Tf-AF647 distribution on the membrane immediately following Tf-AF647 delivery. **d**, Merged image of the channels from (**b**) and (**c**), showing their spatial overlap. Scale bar is 5 μm throughout. Result representative of a recorded series of independent measurements with sample size $n = 13$.

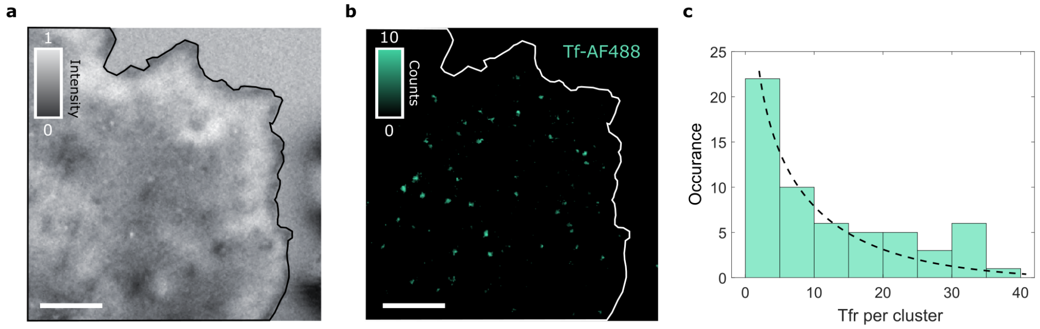

Figure 22: Quantification of the number of TfR labeled within each membrane cluster. **a**, Confocal iSCAT image of the cell region to which μkiss brush labeling of TfR with Tf-AF488 will occur. Black line marks cell boundary for reference. **b**, Fluorescence image from the same membrane region as (**a**), following Tf-AF488 delivery and labeling of TfR clusters. Scale bars in (**a**) and (**b**) are 5 μm. **c**, Histogram showing the calibrated number of TfR per cluster from the image in (**b**).

Next, we consider the properties of our imaging confocal microscope. The volume of the confocal PSF is computed via:

$$V_{\mathrm{PSF}} = \frac{2.33\,\pi^{3/2} n\, w^3}{\mathrm{NA}} \tag{5}$$

where $n = 1.33$ is the refractive index of the medium (water), the numerical aperture $\mathrm{NA} = 1.0$ and w is the e^{-2} beam radius in the xy-plane. We assume a wavelength of 510 nm (peak fluorescence emission of AF488 detected here), and find $V_{\mathrm{PSF}} = 0.561\,\mu\mathrm{m}^{-3}$. We establish a μkiss envelope with concentration $C_{\mathrm{AF488}} = 60220$ molecules $\mu\mathrm{m}^{-3}$ and record the signal intensity with the same acquisition settings as used in the original labeling measurement. We find from within the confocal PSF detection volume a median signal intensity of $I_{\mathrm{det}} = 117$ counts s^{-1} from AF488. This signal is assumed to originate from $V_{\mathrm{PSF}} \times C_{\mathrm{AF488}}$ molecules, that is, $N_{\mathrm{AF488}} = 33790$ molecules. This equates to an average of 3.45×10^{-3} counts s^{-1} per AF488, which is equivalently 0.0138 counts s^{-1} per Tf.

In Fig. 22a we present a confocal iSCAT image of the cell membrane region, that undergoes Tf-AF488 μkiss brush delivery, yielding AF488-Tf labeled TfR clusters shown in Fig. 22b. Using our calibration, we convert the measured optical signal for AF488 in Fig. 22b, assuming that each distinct cluster is well approximated as originating from a single PSF detection volume. In Fig. 22c we plot the distribution of the calculated number of TfR-per-cluster from Fig. 22b using our signal-to-molecule number calibration.

10 Latrunculin Insult

Lat-A application to the targeted cell in Fig. 5 of the main report results in the inward retraction of the cell corpus, shown in Supplementary Video 5. This is manifested in the reduction of the surface area the cell occupies on the substrate. In Fig. 23, we visualize the changing substrate area occupied by the cell that results from application of Lat-A over the full course of the measurement depicted in Fig. 5 (main report).

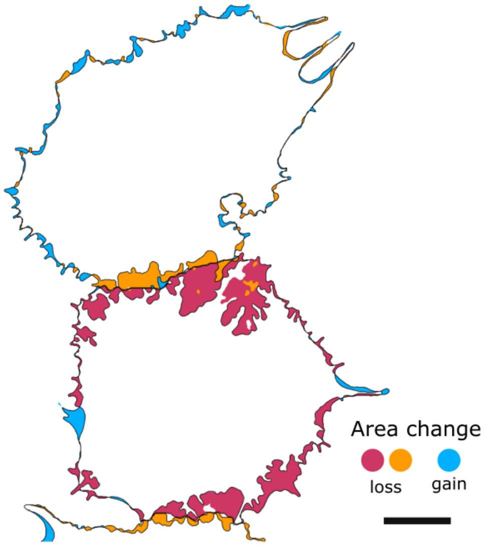

Figure 23: Change in surface area occupied by the targeted and neighboring cells, following Lat-A delivery. White denotes an unchanged area covered by the cells prior to, and following Lat-A delivery. Red and orange denote regions where cell area is lost following Lat-A delivery for the targeted and neighboring cells, respectively. Different colors are used for clarity. Blue regions denote any gain in area covered following Lat-A delivery. Scale bar denotes 5 μm.

11 Reagent Delivery from a Single Sub-micron Micropipette

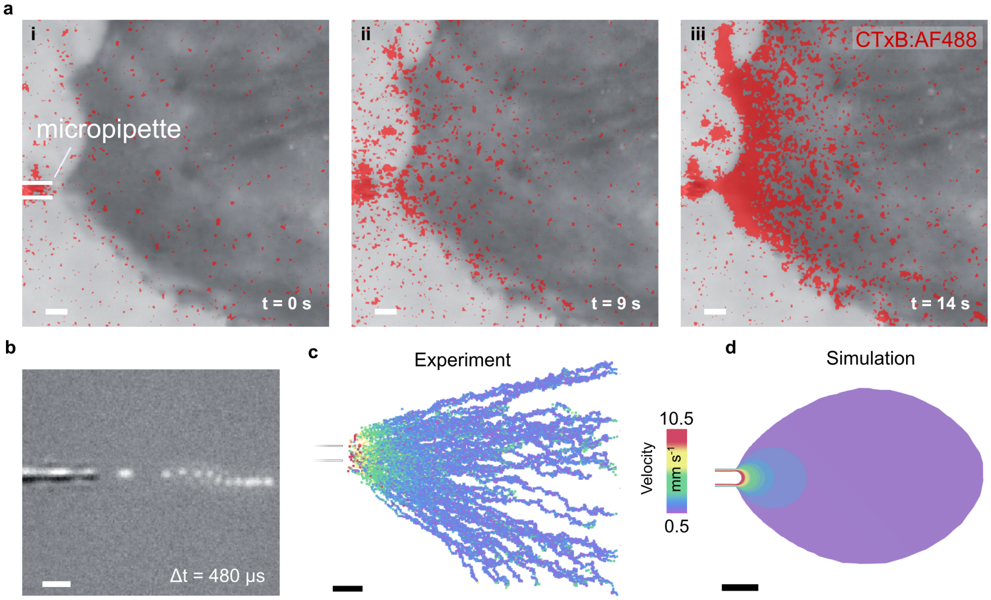

Figure 24: Sub-cellular labeling with a single sub-micron micropipette. **a**, Cell periphery imaged via iSCAT microscopy (grayscale) prior to labeling with CTxB-AF488, shown in red, administered from a micropipette with a 500 nm aperture, (**i**). Following 9 s of continued injection (P = 23 mbar), labeled GM1 on the plasma membrane becomes apparent, (**ii**). Following 14 s of continuous injection, a broad peripheral region is labeled, and injection thereafter ceased, (**iii**). **b**, Widefield iSCAT time-lapse image showing an 80 nm gold nanoparticle ejected out of a 500 nm-aperture micropipette. **c**, A composite image showing 100 trajectories of ejected nanoparticles, with the color scale denoting instantaneous velocity. **d**, Numerical modeling of the fluid velocity field for the micropipette shown in (**c**), with which the color scale is shared. Scale range is restricted to 0.5 - 10.5 mm s^{-1} for clarity. Scale bar throughout is 1 μm.

To illustrate the advantages inherent to μkiss delivery over traditional labeling strategies, we performed an analogous measurement to that presented in Fig. 3b of the main text, with the same objective of achieving micro-scale labeling of the plasma membrane of the live cell, using a single injecting micropipette with a sub-micron-sized aperture. A Femtotip II micropipette from Eppendorf was used, which features an aperture of 500 nm. Again, a 1 mM CTxB-AF488 reagent solution was used for the purpose of labeling the GM1 molecules within the membrane of the cell. The micropipette aperture was positioned in the vicinity of the plasma membrane, but not in contact (comparable to that used for the μkiss labeling), and flow actuated at the lowest pressure wherein reagent administration could be discerned (corresponding to 20 mbar). For the purpose of this measurement, a more sensitive microscope was used; a Nikon Confocal microscope was used with an oil-immersion NA = 1.4 objective achieving single-molecule detection sensitivity (verified by observation of single-molecule bleaching).

Figure 24a,i-iii shows excerpts from the image sequence for GM1 labeling recorded via confocal iSCAT imaging of the cell and micropipette (shown in grayscale) and the CTxB-AF488 reagent (presented in red). One observes the area labeled corresponds to a broad area of the cell membrane periphery that intersects the jet of fluid injected by the micropipette. Secondly, although the reagent solution was of the same concentration as used in Fig. 3a

(main report), with improved sensitivity of detection used here, a labeling time of many seconds is required for a substantial signal to accumulate from the labeled GM1 (with the peak signal occurring after 14 s of labeling).

The inability for single-channel micropipettes to match the spatial resolution of μkiss labeling is caused by the wide jet of unconfined fluid that emanates out of the micropipette aperture when not in contact with the target surface. Even under gentle operating conditions used here, the jet extends many microns outward. To visualize this jet, we again performed high-speed particle tracking (25,000 fps) of 80 nm gold nanoparticles similarly loaded into the 500 nm-aperture micropipette. Figure 24b presents a composite time-lapse of high-speed imaging of a gold nanoparticle being injected out of the micropipette. Each frame was exposed for 5 μs and a frame every 480 μs is shown. The trajectories of 100 particles are shown in Fig. 24c, wherein the jet profile out of the 500 nm aperture is clearly visible. Our tracking reveals a peak particle velocity of 11 $\mathrm{mm\,s^{-1}}$ - hence larger than that used for μkiss. We verified our experimental observations of fluid velocity with numerical simulation under the same conditions, which shows strong agreement, presented in Fig. 24d.

References

[1] Kenneth T. Brown and Dale G. Flaming. New microelectrode techniques for intracellular work in small cells. *Neurosci.*, 2:813–827, 1977.

[2] Yi-Teng Hsiao, Tsai-Ying Wu, Bo-Kuan Wu, Shi-Wei Chu, and Chia-Lung Hsieh. Spinning disk interferometric scattering confocal microscopy captures millisecond timescale dynamics of living cells. *Opt. Expr.*, 30:45233–45245, 2022.

[3] Michelle Küppers, David Albrecht, Anna D. Kashkanova, Jennifer Lühr, and Vahid Sandoghdar. Confocal interferometric scattering microscopy reveals 3D nanoscopic structure and dynamics in live cells. *Nat. Commun.*, 14:1962, 2023.

[4] Richard W. Taylor, Reza Gholami Mahmoodabadi, Verena Rauschenberger, Andreas Giessl, Alexandra Schambony, and Vahid Sandoghdar. Interferometric scattering microscopy reveals microsecond nanoscopic protein motion on a live cell membrane. *Nat. Photon.*, 13:480–487, 2019.

[5] Anna Kashkanova, Martin Blessing, André Gemeinhardt, Didier Soulat, and Vahid Sandoghdar. Precision size and refractive index analysis of weakly scattering nanoparticles in polydispersions. *Nat. Meth.*, 19:586–593, 2022.

[6] Minchul Kang, Charles A. Day, Anne K. Kenworthy, and Emmanuele DiBenedetto. Simplified equation to extract diffusion coefficients from confocal frap data. *Traffic*, 13:1589–1600, 2012.

[7] Magnus Röding, Leander Lacroix, Annika Krona, Tobias Gebäck, and Niklas Lorén. A highly accurate pixel-based FRAP model based on spectral-domain numerical methods. *Biophys. J.*, 116:1348–1361, 2019.

[8] Erik M. M. Manders, Fons J. Verbeek, and Jacob A. Aten. Measurement of co-localization of objects in dual-colour confocal images. *J. Microsc.*, 169:375–382, 1993.
