# Supplementary material for: A paintbrush for delivery of nanoparticles and molecules to live cells with precise spatiotemporal control
Source: Nat Methods. 2024 Feb 12;21(3):512–20. doi: 10.1038/s41592-024-02177-x (PMC10927540; doi:10.1038/s41592-024-02177-x)
Supplement: Supplementary file 2 — Reporting Summary [file 41592_2024_2177_MOESM2_ESM.pdf]

nature portfolio

Corresponding author(s): Vahid Sandoghdar

Last updated by author(s): Dec 22, 2023

Reporting Summary

Nature Portfolio wishes to improve the reproducibility of the work that we publish. This form provides structure for consistency and transparency in reporting. For further information on Nature Portfolio policies, see our Editorial Policies and the Editorial Policy Checklist.

Statistics

For all statistical analyses, confirm that the following items are present in the figure legend, table legend, main text, or Methods section.

n/a	Confirmed	
☐	☒	The exact sample size (*n*) for each experimental group/condition, given as a discrete number and unit of measurement
☐	☒	A statement on whether measurements were taken from distinct samples or whether the same sample was measured repeatedly
☒	☐	The statistical test(s) used AND whether they are one- or two-sided<br>*Only common tests should be described solely by name; describe more complex techniques in the Methods section.*
☒	☐	A description of all covariates tested
☒	☐	A description of any assumptions or corrections, such as tests of normality and adjustment for multiple comparisons
☐	☒	A full description of the statistical parameters including central tendency (e.g. means) or other basic estimates (e.g. regression coefficient) AND variation (e.g. standard deviation) or associated estimates of uncertainty (e.g. confidence intervals)
☒	☐	For null hypothesis testing, the test statistic (e.g. *F*, *t*, *r*) with confidence intervals, effect sizes, degrees of freedom and *P* value noted<br>*Give P values as exact values whenever suitable.*
☒	☐	For Bayesian analysis, information on the choice of priors and Markov chain Monte Carlo settings
☒	☐	For hierarchical and complex designs, identification of the appropriate level for tests and full reporting of outcomes
☐	☒	Estimates of effect sizes (e.g. Cohen's *d*, Pearson's *r*), indicating how they were calculated

Our web collection on statistics for biologists contains articles on many of the points above.

Software and code

Policy information about availability of computer code

Data collection	Zeiss Zen 3.0 was used for recording confocal fluorescence and confocal iSCAT data. Phantom Camera Control (PCC) was used for recording highspeed single particle tracking data via iSCAT microscopy. COMSOL Multiphysics 6.0 was used for numerical simulations.
Data analysis	Data analysis comprises image processing, parameter extraction and also fitting of functions to data distributions (i.e. fitting of a 2D Gaussian). This was performed using MATLAB 2022a using standard in-built functions.

For manuscripts utilizing custom algorithms or software that are central to the research but not yet described in published literature, software must be made available to editors and reviewers. We strongly encourage code deposition in a community repository (e.g. GitHub). See the Nature Portfolio guidelines for submitting code & software for further information.

Data

Policy information about availability of data

All manuscripts must include a data availability statement. This statement should provide the following information, where applicable:

- Accession codes, unique identifiers, or web links for publicly available datasets
- A description of any restrictions on data availability
- For clinical datasets or third party data, please ensure that the statement adheres to our policy

Considering the large size of the individual raw videos, the datasets are available from the corresponding author on request. Requests will be answered within three

weeks. Source data are provided with this paper. Source data relating to Figure 1 is available via https://doi.org/10.5281/zenodo.10419371.

Human research participants

Policy information about studies involving human research participants and Sex and Gender in Research.

Reporting on sex and gender	No human participants were involved in this study.
Population characteristics	Not applicable
Recruitment	Not applicable
Ethics oversight	Not applicable

Note that full information on the approval of the study protocol must also be provided in the manuscript.

Field-specific reporting

Please select the one below that is the best fit for your research. If you are not sure, read the appropriate sections before making your selection.

☒ Life sciences ☐ Behavioural & social sciences ☐ Ecological, evolutionary & environmental sciences

For a reference copy of the document with all sections, see nature.com/documents/nr-reporting-summary-flat.pdf

Life sciences study design

All studies must disclose on these points even when the disclosure is negative.

Sample size	In our study, we present our technology on a number of representative experimental scenarios, that did not require specific sample sizes. No claims were made regarding biological significance. All experiments were at least performed multiple times, each on different individual cells. In the case of diffusion studies on SLBs, FRAP and µkiss brushing were performed 30 times on the same membrane. To avoid variations between different SLB preparations, the same preparation material was used to form a bilayer for both the FRAP and µkiss measurements.
Data exclusions	Representative data is shown for each experiment. No data was excluded.
Replication	All data presented in this manuscript is a fair representation of experiments performed. All measurements have been repeated several times with different rounds of sample preparation. We obtained similar results for all experiments, with low statistical variation. In Fig. 1e, particle tracking was performed on 200 nanoparticles from one single experimental run, and all trajectories are shown in the figure panel. This measurement was repeated separately three times using different micropipettes (n = 3). In Fig. 2, both FRAP and µkiss were separately performed on the same bilayer membrane for each measurement. Both bilayer membranes were produced from the same sample preparation. Each experiment was ran for a total of 30 measurements each (n = 30). Fig. 2c present data from one measurement, and Fig. 2e presents all 30 measured diffusion constants from both measurement modalities (n = 30). In Fig. 3b, punctual plasma membrane labeling was performed a total of 25 times on different cells from the same culture sample (n = 25). The analogous FRAP measurement was also performed on a total of eight different cells from the same cell culture sample (n = 8). In Fig. 3e, demecolcine µkiss-delivery was performed once on a single cell, and repeated on a different cell for a total of five cells (n = 5). In Fig. 3j, nanoplastic particles were µkiss-delivered to a single COS-7 cell, and repeated on a different cell for a total of three cells (n = 3). In Fig. 3k, VLPs were µkiss-delivered to a single COS-7 cell, and repeated on a different cell for a total of three cells (n = 3). In Fig. 4, Tf-AF647 was µkiss-delivered to a single COS-7, and repeated on a different cell for a total of 13 cells (n = 13). In Fig. 5, Lat-A was µkiss-delivered to a single COS-7, and repeated on a different cell for a total of two times (n = 2).
Randomization	Randomization was not required to avoid bias during data analysis.
Blinding	Not required; all measurements of the same kind were analysed using the same exact code without modification. In this regard, the analysis is blind.

Reporting for specific materials, systems and methods

We require information from authors about some types of materials, experimental systems and methods used in many studies. Here, indicate whether each material, system or method listed is relevant to your study. If you are not sure if a list item applies to your research, read the appropriate section before selecting a response.

Materials & experimental systems

n/a	Involved in the study
☒	☐ Antibodies
☐	☒ Eukaryotic cell lines
☒	☐ Palaeontology and archaeology
☒	☐ Animals and other organisms
☒	☐ Clinical data
☒	☐ Dual use research of concern

Methods

n/a	Involved in the study
☒	☐ ChIP-seq
☒	☐ Flow cytometry
☒	☐ MRI-based neuroimaging

Eukaryotic cell lines

Policy information about cell lines and Sex and Gender in Research

Cell line source(s)	COS-7 cells were obtained from the DSMZ (German Collection of Microorganisms and Cell Cultures GmbH).
Authentication	Cells were routinely validated through morphology. As the cells were obtained commercially, no further authentication was performed.
Mycoplasma contamination	Cells were routinely tested negative for mycoplasma contamination.
Commonly misidentified lines (See ICLAC register)	No commonly misidentified cell lines were used in our study.
